# Available cloned genes and markers for genetic improvement of biotic stress resistance in rice

**DOI:** 10.3389/fpls.2023.1247014

**Published:** 2023-09-05

**Authors:** Eliza Vie Simon, Sherry Lou Hechanova, Jose E. Hernandez, Charng-Pei Li, Adnan Tülek, Eok-Keun Ahn, Jirapong Jairin, Il-Ryong Choi, Raman M. Sundaram, Kshirod K. Jena, Sung-Ryul Kim

**Affiliations:** ^1^ Rice Breeding Innovation Department, International Rice Research Institute (IRRI), Laguna, Philippines; ^2^ Institute of Crop Science (ICropS), University of the Philippines Los Baños, Laguna, Philippines; ^3^ Taiwan Agricultural Research Institute (TARI), Council of Agriculture, Taiwan; ^4^ Trakya Agricultural Research Institute, Edirne, Türkiye; ^5^ National Institute of Crop Science, Rural Development Administration (RDA), Republic of Korea; ^6^ Division of Rice Research and Development, Rice Department, Bangkok, Thailand; ^7^ ICAR-Indian Institute of Rice Research, Rajendranagar, Hyderabad, India; ^8^ School of Biotechnology, KIIT Deemed University, Bhubaneswar, Odisha, India

**Keywords:** biotic stress, marker-assisted selection, brown planthopper, blast, bacterial blight, marker, rice

## Abstract

Biotic stress is one of the major threats to stable rice production. Climate change affects the shifting of pest outbreaks in time and space. Genetic improvement of biotic stress resistance in rice is a cost-effective and environment-friendly way to control diseases and pests compared to other methods such as chemical spraying. Fast deployment of the available and suitable genes/alleles in local elite varieties through marker-assisted selection (MAS) is crucial for stable high-yield rice production. In this review, we focused on consolidating all the available cloned genes/alleles conferring resistance against rice pathogens (virus, bacteria, and fungus) and insect pests, the corresponding donor materials, and the DNA markers linked to the identified genes. To date, 48 genes (independent loci) have been cloned for only major biotic stresses: seven genes for brown planthopper (BPH), 23 for blast, 13 for bacterial blight, and five for viruses. Physical locations of the 48 genes were graphically mapped on the 12 rice chromosomes so that breeders can easily find the locations of the target genes and distances among all the biotic stress resistance genes and any other target trait genes. For efficient use of the cloned genes, we collected all the publically available DNA markers (~500 markers) linked to the identified genes. In case of no available cloned genes yet for the other biotic stresses, we provided brief information such as donor germplasm, quantitative trait loci (QTLs), and the related papers. All the information described in this review can contribute to the fast genetic improvement of biotic stress resistance in rice for stable high-yield rice production.

## 1 Introduction

Rice (*Oryza sativa* L.) is a staple food of more than 50% of the world’s population; notably, it is the most important crop in Asian countries. Recently, rice consumption has been rapidly increasing in Africa as well (Seck et al., 2012). Stable high-yield production of rice is highly associated with global food security (Bandumula, 2018). However, rice plants are inevitably encountering pressing challenges from different types of biotic/abiotic stresses that cause significant rice grain yield reduction (Khush, 2005; Dixit et al., 2020). Biotic stresses caused by pests and diseases pose a significant risk to global rice yield production by 52%, of which approximately 30% of these damages are due to pathogen infection (Savary et al., 2019; Jamaloddin et al., 2021). In addition, global climate change is a major threat to global food security (Schneider and Asch, 2020). A changing climate will influence the distribution and possibly the impact of rice diseases (Bebber, 2015; Chaloner et al., 2021) as well as host and disease interactions, mechanism, reproduction, and survival of pathogens (Velásquez et al., 2018).

Rice plants are attacked by diverse biotic agents, including insect pests, fungal and bacterial pathogens, and viruses. The prevalence of species of pathogens and biotypes/pathotypes is variable based on the environmental condition and geographical locations. Over the past decades, outbreaks due to pests and diseases have caused serious economic damage to rice-growing countries from time to time, locally and globally. For instance, some devastating damage from brown planthopper (BPH) infestation has been reported in different years in many rice-growing countries, including tropical and temperate Asia (Dyck and Thomas, 1979; Jena and Kim, 2010). Rice blast disease causes a loss of rice yield sufficient to feed 60 million people worldwide (Fahad et al., 2019; Singh et al., 2020). As a viral disease, a series of large-scale outbreaks of tungro were recorded in many tropical Asian countries, and it causes yield losses of 5% to 10% annually (Dai and Beachy, 2009). In Africa, rice yellow mottle virus (RYMV) is one of the most problematic biotic stresses, it reduces grain yield by 10%–100%, and severe attacks can lead to plant death (Kouassi et al., 2005). Still, today, severe biotic stress damage is reported in local or national media, implying that biotic stress damage affects local rice farmers, particularly small and marginal farmers.

There are several practical methods used to control pathogens, such as chemical spraying, crop rotation, field management, and host resistance. Among these, genetic improvement of host resistance by introgression of resistance genes through breeding and cultivation of resistant varieties is the most cost-effective and environmental-friendly strategy for controlling biotic agents. Thus, much effort has been exerted by scientists and breeders in isolating germplasms possessing resistance to a variety of biotic stresses from cultivars, landraces, and wild rice species in the genus *Oryza*. Through genetic analysis, they have also identified the genetic factors (quantitative trait loci (QTLs)/genes) that provide resistance from the isolated germplasm.

Once the genetic factors conferring biotic stress resistance are identified, they can be easily and effectively transferred to the target background varieties by marker-assisted selection (MAS) compared to the conventional phenotype-based selection. DNA markers that can discriminate the alleles (sequences) between the donor and elite susceptible variety play important roles in efficiently deploying the identified genetic factors. Different types of molecular markers have been developed based on the types of sequence variations (short or long InDels and single-nucleotide polymorphisms (SNPs)) and successfully applied in the genetics and breeding of rice. Among them, the PCR-gel-based markers such as simple sequence repeat (SSR) markers, also called rice microsatellite (RM) markers, InDel markers, dominant PCR markers, tetra-primer method markers, and cleaved amplified polymorphic sequence (CAPS: PCR-restriction enzyme application-gel) markers are the most common in rice MAS breeding due to simplicity, in-house accessibility, and easiness to breeders (McCouch et al., 2002; Chen H, et al., 2011; Wang et al., 2012; Kim et al., 2016; Nadeem et al., 2018).

To improve the genetic potential of biotic stress resistance through MAS, two key factors are essential: genetic factors (QTLs and genes) and molecular tools (DNA markers). Compared to the QTL level of genetic factors, the cloned genes/alleles have some advantages: i) the genetic effect will be quite reliable because it was functionally validated by using transgenic approaches such as complementation test, RNAi, and CRISPR tools; ii) the exact physical location of the gene is identified, and thus, it enables a precision marker-assisted introgression of the target gene without linkage drag caused by the neighboring genes. Many biotic stress resistance genes were cloned from cultivars, landraces, and wild rice germplasm possessing “natural variations”, but some of the genes were identified by transgenic approaches such as overexpression, RNAi, and CRISPR and also by using rice T-DNA tagging lines. Several review papers already covered recent advances in understanding the molecular mechanism of biotic stress resistances for BPH (Yan et al., 2023), blast (Liu W, et al., 2013; Li et al., 2019), and bacterial blight (Jiang et al., 2020; Pradhan et al., 2020) and also broad-spectrum disease resistance in rice (Ke et al., 2017; Liu et al., 2021). In this review, we focused on consolidating all the available cloned genes/alleles with corresponding donors possessing “natural variations” and all the related DNA markers for the breeding aspects. In addition, we briefly described some review papers and recent publications about the QTLs or germplasm if the cloned genes are not available for specific pathogens. We aimed to provide breeding-related information so that breeders can easily select the available resistant genes/alleles and the associated markers for the fast deployment of the proper genes/alleles in their breeding programs to deal with stable high-yield rice production and climate change.

## 2 Precision marker-assisted breeding by using the cloned genes/alleles

Deployment of QTLs and genes through marker-assisted breeding has been successfully improving the genetic potential of target traits in many crops. However, occasional acquisition of biotic stress resistance by the breeding process used to be associated with yield penalties in crops (Brown, 2002) and also grain quality in rice (Fukuoka et al., 2009) probably due to the presence of unfavorable genes located in the vicinity of the target biotic stress resistance locus (also called linkage drag). Thus, precise introgression of biotic stress resistance genes through marker-assisted breeding of the cloned genes can reduce unexpected penalties in yield, grain qualities, and also other agronomic traits in the final breeding products. Recent advances in DNA sequencing, genotyping technologies, genome-wide association study (GWAS), functional genomics, and gene validation by using transgenic approaches have been accelerating the identification of the causal genes governing the target traits. Notably, many biotic stress resistance genes from the previously identified major QTLs have been gradually cloned. The cloned genes/alleles possessing natural variations are valuable for the genetic improvement of biotic stress resistance in rice. Furthermore, unlike QTL level genetic factors (more than several hundred kb), breeders can precisely introgress the gene (100 kb) using marker-based recombinant selection to avoid unwanted phenotypes caused by linkage drag in the final breeding lines because the exact physical location of the causal gene is clearly known. To date, 48 genes have been cloned for the major rice biotic stress, including bacterial blight (BB), blast, BPH, and rice viruses. The cloned gene names, gene IDs of rice databases (RAP-DB and MSU), encoding proteins, the physical location of the genes, donor germplasm, and its original research papers are summarized in this review. In some cases, the previously reported major QTLs from different sources were identified as the same gene (same locus) with different alleles (different sequences). For example, *BPH1*=*BPH10*=*BPH18*=*BPH21*/*BPH2*=*BPH26*/*BHP7*/*BPH9* on the long arm of Chr 12 (“=“ and “/” means identical and different alleles, respectively) and *Pi9*/*Pi2*/*Piz-t*/*Pi50*/*PigmR* on the short arm of Chr 6 are the different resistant alleles but the same locus. Due to the same physical locations, those alleles cannot be pyramided, and thus, the potential best allele should be selected and used in the breeding program. In this review, we focused on the cloned biotic stress resistance genes with the gene-linked markers. Moreover, we briefly mentioned some genetic resources such as QTLs or donor materials if there are no cloned genes yet for some biotic stresses.

## 3 Insect pests and available genetic resources

Globally, more than 100 species of insects attack rice plants, and approximately 20 of them can cause economic damage (Pathak and Khan, 1994). Major insect pests of rice are stem borers, leafhoppers and planthoppers, gall midges, and grain-sucking bugs. Efforts to isolate the resistant germplasm and genetic factors against insect pests identified a number of QTLs for the major insect pests. At the gene level, a handful of genes were cloned for only BPH resistance, but to date, no genes have been cloned yet for other insect pest resistance. Here, we described BPH resistance genes cloned and some genetic resources (QTLs and donor sources) for other insect pests.

### 3.1 Brown planthopper (*Nilaparvata lugens*)

Among the major insect pests, BPH is one of the most destructive pests, especially in Asian countries including both tropical and temperate zones, causing severe economic loss to the rice crop through directly sucking phloem sap, often causing “hopper burn”, and it serves as a vector for transmission of rice ragged stunt virus (RRSV) and rice grassy stunt virus (RGSV) (Cabauatan et al., 2009). To date, more than 45 genetic loci providing BPH resistance have been identified from diverse plant materials, including cultivars, landraces, and wild rice species. Among them, seven genes (seven independent loci) comprising 10 different alleles for BPH resistance were cloned, that is, *BPH14*, *BPH30*, *BPH17*, *BPH6*, *BPH29*, *BPH32*=*BPH3*, and *BPH1*=*BPH10*=*BPH18*=*BPH21*/*BPH2*=*BPH26*/*BHP7*/*BPH9*. The cloned genes with physical locations, RAPDB/MSU gene ID, protein encoded, donor sources, and corresponding references are summarized in **Table 1**. *BPH14* gene encoding nucleotide-binding site (NBS) and leucine-rich repeats (LRRs), “NBS-LRR” or “NLR” in short, was first cloned from the previously mapped *Qbp1* on Chr 3 of the *Oryza officinalis* introgression by genetic mapping and following transgenic complementation test (Du et al., 2009). With similar approaches, the *BPH17* QTL on Chr 4S of the Sri Lankan rice variety, Rathu Heenati (Sun et al., 2005), revealed that three repeats of lectin receptor kinase gene (*OsLecRK1-OsLecRK3*) are responsible for BPH resistance (Liu et al., 2015). However, Liu et al. (2015) named the gene identified from the *BPH17* QTL as *BPH3* gene, and thus, it might cause confusion with the original *BPH3* QTL mapped on Chr 6S of donors (PTB33 and Rathu Heenati varieties) (Jairin et al., 2007). To avoid confusion, we followed the original *BPH17* QTL name as *BPH17* gene name in this review. Afterward, Ren et al. (2016) cloned the causal gene of BPH resistance from the previously fine-mapped *BPH3* locus of PTB33 (Jairin et al., 2007) using bioinformatics and transgenic validation experiments. The cloned gene encodes an unknown short consensus repeat (SCR) domain-containing protein and the *BPH3* QTL was renamed as *BPH32* (*BPH32*=*BPH3*) (Ren et al., 2016). Some of the BPH-resistant loci from different sources overlapped at the same locus, resulting in four clusters on chromosomes 4S, 4L, 6S, and 12L (Fujita et al., 2013; Du et al., 2020). From the largest BPH QTL cluster on Chr 12L containing *BPH1*, *BPH2*, *BPH7*, *BPH9*, *BPH10*, *BPH18*, *BPH21*, and *BPH26* (Fujita et al., 2013), *BPH26* encoding NBS-LRR protein was first cloned from the *BPH26* QTL derived from ADR52 (Tamura et al., 2014). Then, *BPH18* from the *BPH18* QTL originated from the *Oryza australiensis* introgression line (IL) (IR65482-7-216-1-2) was cloned and identified as the same gene with *BPH26* because physically two genes are located at the same locus on Chr 12L. However, the sequences, including promoter and protein-coding sequences (CDS) and also BPH reactions, were different between *BPH26* and *BPH18* (Ji et al., 2016). *BPH9* derived from Pokkali was also identified as the same gene as *BPH18*/*BPH26*, but it showed different gene sequences and also different BPH reactions (Zhao et al., 2016), suggesting that all three are the same gene (locus) but functionally different alleles. Based on the sequence analysis of the Chr 12L BPH cluster, Zhao et al. (2016) classified the eight genes into four allelotypes, *BPH1*=*BPH10*=*BPH18*=*BPH21*/*BPH2*=*BPH26*/*BHP7*/*BPH9*. However, the BPH near-isogenic lines (NILs) with the same allele types (*BPH10*, *BPH18*, and *BPH21*) showed slightly different BPH resistance among the same allele types (Jena et al., 2017). Although four different functional alleles were identified on Chr 12L, they cannot be pyramided by MAS breeding due to their same locations. Guo et al. (2018) cloned the *BPH6* encoding NBS-LRR protein from the previously found *BPH6* QTL originating from the Swarnalata variety, which exhibits resistance to biotype 4, the most devastating BPH biotype in South Asia, of Bangladesh BPH populations (Kabish and Khush, 1988). The recessive gene *BPH29* located at Chr 6 was found to encode a B3-domain containing protein from the RBPH54 IL possessing BPH resistance derived from the wild rice species *Oryza rufipogon* (Wang Y, et al., 2015). *BPH30* gene located on Chr 4 of the *indica* variety AC-1613 was identified as a gene that encodes a novel protein with two leucine-rich domains (Shi et al., 2021). In addition to the cloned BPH genes, a number of QTLs and fine-mapped QTLs are also available (Fujita et al., 2013; Naik et al., 2018; Du et al., 2020). Moreover, using 10 different BPH genes/QTLs, 25 NILs possessing single or two to three genes were developed in an *indica* variety background, IR24 (Jena et al., 2017). The set of BPH NILs will be useful for screening suitable BPH genes/alleles against regional BPH biotypes and for genetic improvement of BPH resistance in the local elite variety backgrounds. To achieve durable and broad-spectrum resistance, QTL/gene pyramiding approaches are widely used in breeding programs. Overall, the BPH-NILs with two to three genes exhibited more strong and broad-spectrum resistance than the NILs harboring a single BPH gene (Jena et al., 2017). In addition, pyramiding effects of two to three BPH gene combinations such as *BPH14* + *BPH15*, *BPH6* + *BPH12*, and *BPH13* + *BPH14* + *BPH15* were observed in different backgrounds or breeding programs (Hu et al., 2012; Qiu et al., 2012; Hu et al., 2016).

**Table 1:** The cloned BPH resistance genes.

Gene	Chr	Location (bp)^(a)^	MSU_ID	RAPDB_ID	Encoding protein	Resistant/donor allele	Inheritance pattern of R- allele	Reference
*BPH14*	3	35,693,286	Os03g63150	Os03g0848700	NBS-LRR	*Oryza officinalis* IL	Dominant	Du et al., 2009
*BPH30*	4	929,966	Os04g02520	–	Protein with two leucine-rich domains (LRDs)	AC-1613	Dominant	Shi et al., 2021
*BPH17^(b)^*	4	6,940,275	Os04g12540–Os04g12560–Os04g12580	Os04g0201900–Os04g0202300–Os04g0202500	A cluster of three genes encoding plasma membrane-localized lectin receptor kinases (OsLecRK1-OsLecRK3)	Rathu Heenati	Dominant	Liu et al., 2015
*BPH6*	4	21,396,879	Os04g35210	Os04g0431700	Atypical LRR	Swarnalata	Dominant	Guo et al., 2018
*BPH29*	6	484,346	Os06g01860	Os06g0107800	B3 domain-containing protein	RBPH54 (*Oryza rufipogon* IL)	Recessive	Wang Y, et al., 2015
*BPH32* =*BPH3^©^*	6	1,223,069	Os06g03240	Os06g0123200	Unknown short consensus repeat (SCR) domain-containing protein	Ptb33	Dominant	Ren et al., 2016
*BPH1*=*BPH10*=*BPH18*=*BPH21*/*BPH2*=*BPH26*/*BHP7*/*BPH9^(d)^*	12	22,886,341	Os12g37290	Os12g0559400	NBS-LRR	IR65482-7-216-1-2 (*BPH18*), ADR52 (*BPH26*), T12 (*BPH7*), Pokkali (*BPH9*)	Dominant	Tamura et al., 2014 (*BPH26*), Ji et al., 2016 (*BPH18*), Zhao et al., 2016 (*BPH9* and other alleles)

“=“ means the identical allele, and “/” means the different alleles at the same locus.

^(a)^Location of the translation start codon (ATG) of the cloned genes on the rice reference genome IRGSP1.0 (https://rapdb.dna.affrc.go.jp/).

^(b)^BPH17 was identified from the mapping populations derived from the cross Rathu Heenati (R) and 02428 variety (S) by Sun et al. (2005). In a subsequent study, Liu et al. (2015) cloned the BPH resistance gene from the same materials, but the gene was probably mistakenly named BPH3 in the publication. Hence, to avoid confusion with previously reported BPH3 QTL (Jairin et al., 2007), the original name QTL name (BPH17) was given in this review.

^(c)^BPH32 was identified by using bioinformatics and transgenic gene validation experiments by Ren et al. (2016) from the previously fine-mapped BPH3 locus (Jairin et al., 2007).

^(d)^Eight BPH genes clustered on Chr 12L were identified as multi-alleles with four different sequences (four allele types) at the same locus (Zhao et al., 2016). However, the NILs with the same allele types (BPH10, BPH18, and BPH21) showed a bit different BPH resistance among the same allele types (Jena et al., 2017).

### 3.2 Other planthoppers

A handful of genetic factors governing resistance against planthoppers, including small brown planthopper (SBPH: *Laodelphax striatellus*), white-backed planthopper (WBPH: *Sogatella furcifera*), green leafhopper (GLH: *Nephotettix virescens*), and green rice leafhopper (GRH: *Nephotettix cincticeps*), have been identified from diverse germplasms and are well summarized in a few review papers (Fujita et al., 2013; Du et al., 2020). In this review, we only included recent progress on genetic factors to other planthoppers. A stable locus showing WBPH resistance in 2 years was found in the RM280-RM6909 region on Chr 4L from the Cheongcheong variety (Kim et al., 2021). The high resistance locus designated as *Bph38* to both BPH and WBPH was identified from *O. rufipogon* and was fine-mapped to a 79-kb region on Chr 4 (Yang et al., 2020). Phi et al. (2019) identified a major QTL (*qGRH4.2*=*GRH6*) conferring GRH resistance from a wild species (*Oryza nivara*_IRGC105715) and fine-mapped the locus to ~31-kb region on Chr 4. Recent studies showed a possibility that increasing resistance to multiple insects could be achieved by the pyramiding of insect resistance loci. For example, both GLH and GRH resistance was obtained by pyramiding of two GRH resistance genes, *GRH2* and *GRH4* (Horgan et al., 2018); enhanced resistance against multiple herbivore species, including zig-zag leafhopper (*Recilia dorsalis*), BPH, and WBPH, was shown by pyramiding of two to three GRH resistance loci (*GRH2* and *GRH4-6*) (Horgan et al., 2019).

### 3.3 Rice gall midge (*Orseolia oryzae*)

To date, 12 potential genetic factors (*Gm1*–*Gm12*) conferring resistance against Asian rice gall midges (*O. oryzae*) have been reported. Among them, 10, except for *Gm9* and *Gm10*, are mapped on rice chromosomes (Bentur et al., 2016; Leelagud et al., 2020). Although no *Gm* genes have been fully validated by using transgenic approaches, four QTLs were fine-mapped with potential candidate genes: *gm3* (donor: RP2068-18-3-5 breeding line from Velluthacheera) on 560-kb region of Chr4L (Sama et al., 2014), *Gm4* (donor: Abhaya) on 300-kb region of Chr 8 (Divya et al., 2015), *Gm8* (donor: Aganni) on 430-kb region of Chr 8 (Divya et al., 2018), and *gm12* (donor: MN62M) on 345-kb region of Chr 2 (Leelagud et al., 2020). These four QTLs might be useful in a breeding program. However, the donor sources showing resistance against Indian gall midge biotypes, including Velluthacheera (*gm3*), Abhaya (*Gm4*), and Aganni (*Gm8*), were susceptible to all eight Thailand gall midge populations (Leelagud et al., 2020), suggesting that the suitable genetic factors should be selected based on the potential biotypes of insects.

### 3.4 Other insect pests

Five QTLs associated with leaf-folder (*Cnaphalocrocis medinalis*) resistance, with 8.0%–21.1% phenotypic variance explained (PVE), were found from the double haploid population (CJ06 × TN1), and pyramiding of QTLs affected resistance to leaf-folder (Rao et al., 2010). However, reliable genetic factors controlling other insect resistance, including stem borer and grain-sucking bugs, have not been reported yet.

## 4 Fungal diseases and available genetic resources

Several major fungal pathogens threaten stable high-yield rice production. The major fungal diseases of rice are “bakanae disease” (pathogen: *Gibberella fujikuroi*, syn. *Fusarium fujikuroi*), “brown spot” (pathogen: *Cochliobolus miyabeanus*, syn. *Bipolaris oryzae*, *Helminthosporium oryzae*), “narrow brown leaf spot” also called “narrow brown spot” (pathogen: *Sphaerulina oryzina*, syn. *Cercospora janseana*, *Cercospora oryzae*), “false smut” (pathogen: *Ustilaginoidea virens*), “leaf scald” (pathogen: *Microdochium oryzae*), “sheath blight” (pathogen: *Rhizoctonia solani*, syn. *Thanatephorus cucumeris*), “aggregate sheath spot” (pathogen: *Rhizoctonia oryzae-sativae*), “sheath rot” (pathogen: *Sarocladium oryzae*), “stem rot” (pathogen: *Sclerotium oryzae*, syn. *Nakataea oryzae*), and “blast” (pathogen: *Magnaporthe oryzae*, syn. *Pyricularia oryzae*). Among fungal diseases, blast has been intensively studied compared to other fungal diseases. As a result, a handful of blast-resistance genes have been cloned, but no cloned genes are available yet for other fungal diseases.

### 4.1 Blast (pathogen: *M. oryzae*, syn. *P. oryzae*)

Among the fungal diseases, rice blast is the most devastating fungal disease of rice worldwide, causing a serious threat to the world’s food security. The blast pathogen can affect all above-ground parts of a rice plant, including the leaf, collar, node, neck, parts of the panicle, and sometimes the leaf sheath (IRRI Rice Knowledge Bank). Blast disease occurs in 85 countries, and it causes a 10%–35% loss of harvest (Fisher et al., 2012), and the amount of rice damaged by blast annually is sufficient to feed 60 million people worldwide (Pennisi, 2010; Fahad et al., 2019; Singh et al., 2020). There are over 100 blast resistance QTLs/loci identified from diverse germplasm including cultivars, landraces, and wild relatives of rice (Ashkani et al., 2016; Li et al., 2019). The *Pib* (donor: *indica* cultivar Engkatek) and *Pita* (donor: *indica* cultivar Tadukan) were the first cloned blast resistance genes, and both encode NBS-LRR domains predicted to be cytoplasmic proteins (Wang et al., 1999; Bryan et al., 2000). To date, 23 genes (23 independent loci) consisting of ~35 different alleles have been cloned, including three panicle blast resistance genes *Pb1*–*Pb3* (**Table 2**). The cloned genes were distributed across the rice chromosomes except for chromosomes 5, 7, and 10. Chromosomes 6 and 11 harbored four and six blast genes, respectively (*Pi9* alleles, *Pid4*, *Pid3* alleles, and *Pid2* on Chr 6; *Pia* alleles, *Pi54rh* alleles, *Pik* alleles, *Pb1*, *Pb2*, and *Pb3* on Chr 11). Several blast-resistant QTLs were identified at the same location on the short arm of Chr 6 (10.4-Mb region) from different germplasms. Finally, the causal genes were located at the NLR gene-repeated cluster (*Pi9* locus), and they are regarded as the same genes with different alleles (*Pi9*/*Pi2*=*Piz-5*/*Piz-t*/*Pi50*/*Pigm*/*Pizh*). At the *Pi9* locus, two to 13 repeats of NLR gene were laid next to each other, and the blast-resistant donors possessed nine repeats (*Pi9* and *Pi2*) or 13 repeats (*Pigm*) of NLR genes (Deng et al., 2017). There were sequence variations among the alleles of the responsive NLR gene at the *Pi9* locus, and they showed different reactions to the blast strains. In addition to the cloned genes/alleles, one major QTL (*Pi40*) was identified at the *Pi9* locus from the *O. australiensis*-derived IL (IR65482-4-136-2-2) through fine mapping (Jeung et al., 2007). The *Pi40* introgression in Korean and Turkish varieties showed resistance to a wide range of blast strains in Korea and Turkey (Jeung et al., 2007; Beser et al., 2016). Another major cluster was found on Chr 11 (25.2-Mb region) (*Pik* locus) from various donor materials, and they (*Pik*/*Pik-m*/*Pik-p*/*Pi1*/*Pi7*) were identified as allelic (**Table 2**). Interestingly, most of the cloned blast genes encode NBS-LRR (NLR) protein, except for four genes: *bsr-d1* (C2H2-type zinc finger protein), *pi21* (proline-rich protein), *Pid2* (B-lectin receptor kinase), and *Ptr*=*Pita2* (armadillo repeat protein). The majority of blast-resistant donor alleles/genes are dominant except for *pi21*, which is recessive (Liu W, et al., 2013). *Pi21* encodes a proline-rich protein, and the loss-of-function allele from the resistant donor (Owarihatamochi) confers non-race-specific resistance. *pi21* gene was closely linked to the gene providing poor eating quality. However, the genes were successfully separated by recombination between two genes in the breeding lines, and blast resistance with good eating quality was achieved (Fukuoka et al., 2009). Thus, precise introgression with the cloned target genes is able to reduce the presence of unwanted phenotypes in the final breeding products caused by “linkage drag”. Among the cloned blast genes, *Pi50*, *Pizh*, *Pi54rh*, *Pi56*, *Pi64*, *PigmR*, and *Ptr*=*Pita2* alleles were known as broad-spectrum resistance (Liu et al., 2021). A few sets of NILs with blast resistance sources were developed in both *japonica* and *indica* backgrounds: 20 NILs with 11 blast QTLs/genes in *japonica* background Lijiangxintuanheigu (LTH) (Telebanco-Yanoria et al., 2010) and 28 NILs with 14 QTLs/genes in an *indica* background, CO39 (Telebanco-Yanoria et al., 2011). Moreover, both NIL sets were tested by 20 blast isolates collected in the Philippines. Recently, 21 NILs with 18 QTLs/genes in another *indica* background, US-2, were developed, and the NILs were tested with 31 isolates from Asia (Japan, China, the Philippines, Indonesia, Vietnam, Cambodia, Bangladesh, and Laos) and Africa (Nigeria, Kenya, and Benin) (Fukuta et al., 2022). In blast bioassay with the NIL sets above, most of the genes/QTLs showed differential reactions against different isolates, even in the same country collections, suggesting that the selection of suitable blast genes/alleles based on the local pathotypes/isolates is important to develop blast resistant varieties. Among the blast genes used in the NIL development above, NIL-*Pi9* exhibited resistance or moderate resistance to all 31 isolates from Asia and Africa (Fukuta et al., 2022), suggesting that *Pi9* allele might be useful to breed blast-resistant variety across the rice cultivation countries. The sets of NILs and blast screening data against various isolates will be very useful to pathology studies, the selection of suitable genes/alleles against regional isolates, and breeding programs. To achieve durable and broad-spectrum resistance, pyramiding of resistance genes (two or more) in one background is usually used in the breeding program. There are various gene combinations of blast genes that prove the enhanced blast resistance in both *indica* and *japonica* rice against several blast isolates. Two genes–pyramided lines with *Pi37* + *Pid3*, *Pi5* + *Pi54*, *Pi54* +*Pid3*, and *Pigm* + *Pi37* exhibited significantly enhanced resistance and observable additive effects (Jiang et al., 2019). The gene combinations *Pigm* + *Pi1*, *Pigm* + *Pi54*, and *Pigm* + *Pi33* displayed broad-spectrum resistance (Wu et al., 2019). Broad-spectrum blast resistance was also achieved in the temperate *japonica* varieties by pyramiding three to four genes with *Piz*, *Pib*, *Pik*, *Pita*, and *Pita2* (Zampieri et al., 2023). As proven in many previous studies, stacking suitable blast genes/alleles has strong potential to obtain durable and broad-spectrum resistance in the breeding program.

**Table 2:** The cloned blast resistance genes.

Gene	Chr	Location (bp)	MSU_ID	RAPDB_ID	Encoding protein	Resistant/donor allele	Inheritance pattern of R-allele	Reference
*Pit*	1	2,681,220	Os01g05620	Os01g0149500	NBS-LRR	K59	Dominant	Hayashi K, et al., 2010
*Pi64*	1	33,098,082	Os01g57280	Os01g0781200	NBS-LRR	Yangmaogu	Dominant	Ma et al., 2015
*Pi37*	1	33,120,499	Os01g57310	Os01g0781700	NBS-LRR	St. No. 1	Dominant	Lin et al., 2007
*Pish*/*Pi35*	1	33,136,846	Os01g57340	Os01g0782100	NBS-LRR	Nipponbare (*Pish*), Hokkai 188 (*Pi35*)	Dominant	Takahashi et al., 2010 (*Pish*), Fukuoka et al., 2014 (*Pi35*)
*Pib*	2	35,118,769	Os02g57310	Os02g0818500	NBS-LRR	Engkatek	Dominant	Wang et al., 1999
*bsr-d1*	3	18,435,990	Os03g32230	Os03g0437200	C2H2-type zinc finger protein	Digu	Dominant	Li et al., 2017
*pi21*	4	19,835,206	Os04g32850	Os04g0401000	Proline-rich protein	Owarihatamochi	Recessive	Fukuoka et al., 2009
*Pi63*	4	31,554,480	Os04g52970	Os04g0620950	NBS-LRR	Kahei	Dominant	Xu et al., 2014
*Pi9*/*Pi2*=*Piz-5*/*Piz-t*/*Pi50*/*PigmR^(e)^*/*Pizh*	6	10,387,509	Os06g17900	Os06g0286700	NBS-LRR	*Oryza minuta* IL (75-1-127) (*Pi9*), C101A51 (*Pi2*), Toride 1 (*Piz-t*), Er-Ba-Zhan (*Pi50*), Gumei 4 (*PigmR*), ZH11 (*Pizh*)	Dominant	Qu et al., 2006 (*Pi9*), Zhou et al., 2006 (*Pi2* and *Piz-t*), Su et al., 2015 (*Pi50*), Deng et al., 2017 (*PigmR*), Xie et al., 2019 (*Pizh*)
*Pid4*	6	10,435,819	Os06g17950	Os06g0287500	NBS-LRR	Digu	Dominant	Chen et al., 2018
*Pid3*/*Pi25*/*Pid3-I1*	6	13,054,818	Os06g22460	Os06g0330100	NBS-LRR	Digu (*Pid3*), Gumei2 (*Pi25*), MC276 (*Pid3-I1*)	Dominant	Shang et al., 2009 (*Pid3*), Chen J, et al., 2011 (*Pi25*), Inukai et al., 2019 (*Pid3-I1*)
*Pid2*	6	17,160,333	Os06g29810	Os06g0494100	B-lectin receptor kinase	Digu	Dominant	Chen et al., 2006
*Pi36*	8	2,878,884	Os08g05440	Os08g0150150	NBS-LRR	Kasalath (formerly known as Q61)	Dominant	Liu et al., 2007
*Pi5*	9	9,681,913	Os09g15840	Os09g0327600	NBS-LRR	RIL260-Moroberekan	Dominant	Lee et al., 2009
*Pi56*	9	9,777,527	Os09g16000	Os09g0328951	NBS-LRR	Sanhuangzhan No 2 (SHZ2)	Dominant	Liu Y, et al., 2013
*Pia*/*Pi-CO39*	11	6,541,924	Os11g11790–Os11g11810	Os11g0225100–Os11g0225300	Two genes encoding NBS-LRR	Sasanishiki (*Pia*), CO39 (*Pi-CO39*)	Dominant	Okuyama et al., 2011 (*Pia*), Cesari et al., 2013 (*Pi-CO39*)
*Pi54rh*/*Pi54*=*Pik-h*	11	25,263,336	Os11g42010	Os11g0639100	NBS-LRR	*Oryza rhizomatis* (*Pi54rh*), Tetep (*Pi54*)	Dominant	Das et al., 2012 (*Pi54rh*), Zhang et al., 2018 (*Pi54*)
*Pik*/*Pik-m*/*Pik-p*/*Pi1*/*Pi7*	11	27,983,597	Os11g46200-Os11g46210	Os11g0688832–Os11g0689100	Two genes encoding NBS-LRR	Kusabue (*Pik*), Tsuyuake (*Pik-m*), K60 (*Pik-p*), C101LAC (*Pi1*), IRBL7-M (*Pi7*)	Dominant	Zhai et al., 2011 (*Pik*), Ashikawa et al., 2008 (*Pik-m*), Yuan et al., 2011 (*Pik-p*), Hua et al., 2012 (*Pi1*), Gan et al., 2010 (*Pi7*)
*Pita*	12	10,606,359	Os12g18360	Os12g0281300	NBS-LRR	Tadukan	Dominant	Bryan et al., 2000
*Ptr* = *Pita2*	12	10,822,534	Os12g18729	Os12g0285100	Armadillo repeats protein	Katy (*Ptr*), IRBLta2-Re (*Pita2*)	Dominant	Zhao et al., 2018 (*Ptr*), Meng et al., 2020 (*Pita2*)
*Pb1*	11	22,862,447	Os11g38580	Os11g0598500	NBS-LRR	Modan	Dominant	Hayashi N, et al., 2010
*Pb2*	11	27,608,621	Os11g45620	–	NBS-LRR	Jiangnanwan	ND	Yu et al., 2022
*Pb3*	11	27,282,232	Os11g45090	Os11g0675200	NBS-LRR	Haplotype A, Bodao	ND	Ma et al., 2022

ND, not determined.

In contrast to leaf blast resistance, genetic resources for blast disease on other organs/tissues are relatively poor. The first panicle blast resistance gene, *Pb1*, encoding NBS-LRR was cloned from an *indica* cultivar Modan (Hayashi N, et al., 2010). Afterward, it was found that panicle blast resistance by *Pb1* is dependent on at least four other loci (Inoue et al., 2017), suggesting that a level of panicle blast resistance with *Pb1* will be influenced by other genetic factors or background materials. Recently, two additional panicle blast resistance genes, *Pb2* and *Pb3*, were identified through GWAS and validated by transgenic approaches (Ma et al., 2022; Yu et al., 2022). Both genes encode NBS-LRR proteins and are physically close to each other (~360-kb distance between *Pb2* and *Pb3*). Some of the cloned leaf blast genes, such as *Pi25* (Chen J, et al., 2011), *PigmR* (Deng et al., 2017), and *Pid4* (Chen et al., 2018), also showed some level of panicle blast resistance.

### 4.2 Bakanae disease (pathogen: *G. fujikuroi*, syn. *F. fujikuroi*)

To identify the genetic factors governing bakanae disease resistance, QTL mapping and GWAS have been conducted and identified a handful of QTLs on chromosomes 1, 3, 4, 9, and 10 from several different donors, but no genes have been cloned yet. Three major QTLs (*qBK1*, *qBK1.1*, and *qFfR1*) were fine-mapped on the Chr 1 region between 23.32 and 23.67 Mb (Lee et al., 2021).

### 4.3 False smut (pathogen: *U. virens*)

A number of QTLs for false smut resistance have been identified by QTL mapping with bi-parental populations (Andargie et al., 2018; Han et al., 2020; Neelam et al., 2022) and GWAS (Hiremath et al., 2021). The results suggested that false smut resistance seems to quantitate traits governed by multiple genes. Among the QTLs, *qFsr8–1* originated from the Chinese rice landrace MR183–2 and showed the highest PVE (26.0%).

### 4.4 Sheath blight (pathogen: *R. solani*, syn. *T. cucumeris*)

More than 200 QTLs associated with sheath blight (ShB) resistance have been identified from the diverse mapping populations (Zarbafi and Ham, 2019; Goad et al., 2020). Among all the identified ShB QTLs, two loci on Chr 9 (*qShB9-2*) and Chr 11 (*qSBR11-1*) contribute 25% and 14% of PVE, respectively, are the major effect QTLs (Molla et al., 2020), and may be useful in a breeding program.

### 4.5 Brown spot (pathogen: *C. miyabeanus*, syn. *B. oryzae*, *H. oryzae*)

For brown spot (BS) resistance, susceptible and resistant germplasms were identified by several studies. Several cultivars that have been categorized as resistant did not show complete resistance (immunity), but they showed quantitative resistance to BS. To date, more than 20 QTLs with low–mild phenotypic variation (<20%) were identified from several mapping populations, including recombinant inbred lines (RILs), doubled haploid lines (DHLs), and chromosome segment substitution lines (CSSLs) from several different donors (reviewed by Mizobuchi et al., 2016). One major QTL, *qBSR11-kc*, showing 23.0%–25.9% of the total phenotypic variation was identified from *indica* variety CH45 (Matsumoto et al., 2017).

### 4.6 Narrow brown leaf spot also called “narrow brown spot” (pathogen: *S. oryzina*, syn. *C. janseana*, *C. oryzae*)

The genetic architecture of narrow brown spot (narrow brown leaf spot) resistance was almost unknown. A recent genetic analysis using the RIL population derived from the cross between two US varieties (Cypress and LaGrue) identified a single large-effect QTL, *CRSP-2.1*, explaining 81.4% of the phenotypic variation (Addison et al., 2021). The causal gene is not confirmed yet, but the major QTL might be useful in a breeding program.

### 4.7 Aggregate sheath spot (pathogen: *R. oryzae-sativae*)

Aggregate sheath spot disease has been reported in many Asian countries, as well as the USA, South America, and Australia, and it can cause ~20% of yield loss (Lanoiselet et al., 2007). Good levels of resistance to aggregate sheath spot were identified from *O. rufipogon* and successfully transferred into cultivars (McKenzie et al., 1994). Recent GWAS with tropical *japonica* and *indica* populations identified a handful of QTLs (Rosas et al., 2018).

### 4.8 Sheath rot (pathogen: *S. oryzae*)

Rice sheath rot diseases are found in most rice-growing areas of the world and cause 20%–85% ranges of yield losses, making it an emerging ubiquitous destructive disease of rice (Bigirimana et al., 2015). However, rice sheath rot is less studied, and no reliable germplasm or genetic factors have been identified yet.

### 4.9 Stem rot (pathogen: *S. oryzae*, syn. *N. oryzae*)

Stem rot disease resistance was found in wild rice species (*O. nivara* and *O. rufipogon*) and weedy rice (*O. sativa* f. *spontanea*) (Figoni et al., 1983), and the stem rot resistance was successfully transferred from *O. rufipogon* to California rice cultivars through interspecific hybridization (Oster, 1992). Recently, several QTLs for stem rot resistance were identified from *indica* germplasm through a GWAS analysis (Rosas et al., 2018).

## 5 Bacterial diseases and available genetic resources

Rice productions are significantly affected by several major bacterial diseases: BB (pathogen: *Xanthomonas oryzae* pv. *oryzae* (*Xoo*)), “bacterial leaf streak” (BLS) (pathogen: *X. oryzae* pv. *oryzicola* (*Xoc*)), “bacterial sheath brown rot” also called “rice sheath rot” (pathogen: *Pseudomonas fuscovaginae*), and “bacterial seedling rot” (BSR), and “bacterial grain rot” (BGR) caused by the same pathogen (*Burkholderia glumae*). To date, a handful of genes have been cloned for BB resistance, but none yet for other bacterial diseases. Here, we described BB resistance genes cloned and some genetic resources for other bacterial pathogens.

### 5.1 Bacterial blight (pathogen: *X. oryzae* pv. *oryzae* (*Xoo*))

**Figure 1:**
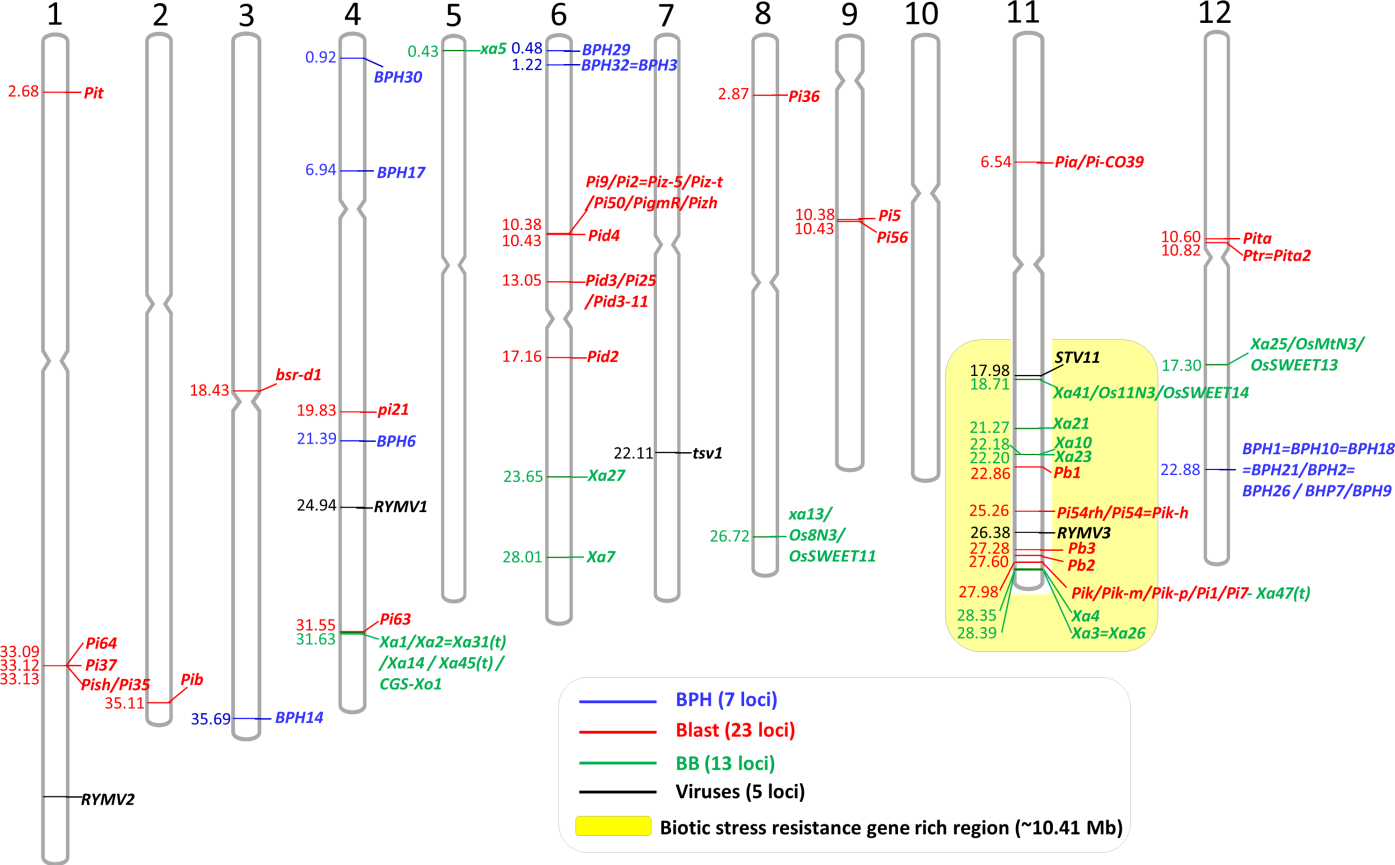
Physical locations of the 48 cloned genes conferring biotic stress resistance in rice. The cloned genes were mapped on the rice reference genome (Os-Nipponbare-Reference-IRGSP-1.0). Blue, red, green, and black bars mean brown planthopper (BPH), blast, bacterial blight, and virus resistance genes, respectively. Biotic stress resistance gene-rich region was highlighted by yellow background (out of 48 genes, 14 genes were on the 10.41-Mb region of the long arm of Chr 11).

Among the bacterial diseases, BB caused by *Xoo* is the most destructive bacterial disease in rice. Thus, it has been intensively studied for the isolation of BB-resistant germplasm, genetic analysis, gene identification, and molecular mechanism of wars between *Xoo* and rice. To date, at least 47 *Xoo* resistance QTLs and genes (named *Xa* genes) have been identified from diverse germplasms, including cultivated rice, rice mutant lines, and wild rice species. *Xa21* from *Oryza longistaminata* introgression line (IRBB21) was first cloned in 1995 by Song et al. and followed by *Xa1* from the IRBB1 line (Yoshimura et al., 1998). Later, *Xa2*, *Xa31(t)*, *CGS-Xo1*, *Xa14*, and *Xa45(t)* were identified as a group of *Xa1* allelic R genes (Ji et al., 2020). Currently, 13 different genes/loci consisting of ~23 allelotypes have been cloned and characterized (**Table 3**), that is, *Xa1*/*Xa2*=*Xa31(t)*/*Xa14*/*Xa45(t)*/*CGS-Xo1*, *Xa3*=*Xa26*, *Xa4*, *xa5*, *Xa7*, *Xa10*, *xa13*/*OsSWEET11*/*Os8N3*, *Xa21*, *Xa23*, *Xa47(t)*, *xa25*/*OsSWEET13*/*OsMtN3*, *Xa27*, and *xa41(t)*/*OsSWEET14*/*Os11N3*. The 13 cloned BB resistance genes encode several types of proteins: NBS-LRR (*Xa1*/*Xa1* alleles and *Xa47(t)*), leucine-rich repeat receptor-like kinases (LRR-RLKs) (*Xa3*=*Xa26* and *Xa21*), a cell wall-associated kinase (WAK) (*Xa4*), executor R proteins (*Xa7*, *Xa10*, *Xa23*, and *Xa27*), SWEET/sugar transporter proteins (*xa13*/*OsSWEET11*, *xa25*/*OsSWEET13*, and *xa41(t)*/*OsSWEET14*), and a transcription factor gamma subunit protein (*xa5*). The genes encoding NBS-LRR, LRR-RLK, and WAK are involved in pathogen recognition and activation of the innate immune system, whereas the genes encoding executor R proteins are transcriptionally activated by the *Xoo* transcription activator-like (TAL) effector protein and trigger programmed cell death (PCD)-based hypersensitive response (HR). Thus, for the genes mentioned above, the functional alleles from the BB-resistant donor sources are dominant. In contrast, BB resistance is caused by sequence mutations at the TAL effector binding sites in the promoter of the SWEET (Sugar Will Eventually be Exported Transporter) genes and thus a recessive allele. BB resistance of *xa5* gene relies on one amino acid difference between resistance and susceptible lines in *Xa5* protein (a general eukaryotic transcription factor), and the BB-resistant allele is recessive (Iyer and McCouch, 2004). The cloned 13 genes are distributed on six chromosomes (one gene each on Chr 4, 5, 8, and 12; two genes on Chr 6; six genes on Chr 11) (**Table 3**, **Figure 1**). Six cloned genes on Chr 11 are closely located to each other in ~10.2-Mb size (18.2–28.4-Mb region on Chr 11) (**Figure 1**). Thus, in the case of gene pyramiding using the six genes on Chr 11, breeders need to consider producing enough progenies for obtaining pyramided alleles that occur by recombination between two closely located genes. Several cloned genes, including *Xa7*, *Xa23*, *xa41*, and *Xa47*, were reported as broad-spectrum resistance genes/alleles (Liu et al., 2021). NILs with single BB resistance genes were developed through IRRI-Japan collaboration designated as “IRBB” lines (Ogawa et al., 1991), and additional NILs (IRBB) with single or multiple BB resistance genes (two to five genes) were developed in the BB-susceptible background IR24 at IRRI, Philippines. Differential reactions of the NILs (IRBB lines) with single and pyramided *Xa* genes to 11 races in the Philippines were observed, and the results are available at the IRRI Rice knowledge bank (http://www.knowledgebank.irri.org/ricebreedingcourse/Breeding_for_disease_resistance_Blight.htm). The IRBB lines possessing multiple *Xa* genes (two to five genes) exhibited broad-spectrum resistance than the single gene introgression IRBB lines. Similarly, pyramiding of *Xa* genes such as *Xa21* + *Xa33*, *Xa21* + *xa13* + *xa5*, and *Xa4* + *xa5* + *Xa7* + *xa13* + *Xa21* offers greater and broader resistance to *Xoo* than an individual resistance gene (Pradhan et al., 2015; BalachIranjeevi et al., 2018; Hsu et al., 2020). The IRBB sets were also tested with 16 isolates in Korea, and the results showed that *xa5* was strong and broad-spectrum resistant than any other *Xa* genes (Jeung et al., 2006). Rice possessing *Xa7* exhibited less disease than lines without *Xa7* over 11 years in the Philippines, even though the virulence of *Xoo* field populations increased. In addition, *Xa7* restricted disease more effectively at high temperatures, while other *Xa* genes were less effective at high temperatures (Webb et al., 2010). The IRBB lines and stacked information including gene reactions, spectrum, durability, and influence of environments will be useful to select suitable genes/alleles for regional/local breeding programs and also for the development of durable and broad-spectrum resistant rice varieties.

**Table 3:** The cloned bacterial blight resistance genes.

Gene	Chr	Location (bp)	MSU_ID	RAPDB_ID	Encoding protein	Resistant/donor allele	Inheritance pattern of R-allele	Reference
*Xa1*/*Xa2*=*Xa31(t)*/*Xa14*/*Xa45(t)*/*CGS-Xo1*	4	31,638,099	Os04g53120	Os04g0622600	NBS-LRR	IRBB1 (*Xa1*), IRBB2 (*Xa2*), IRBB14 (*Xa14*), Zhachanglong (*Xa31(t)*), Carolina Gold Select (*CGS-Xo1*), *Oryza nivara* IRGC102463 (*Xa45(t)*)	Dominant	Yoshimura et al., 1998; Ji et al., 2020
*xa5*	5	437,043	Os05g01710	Os05g0107700	Transcription factor IIA gamma subunit	IRBB5	Recessive	Blair et al., 2003; Iyer and McCouch, 2004
*Xa27*	6	23,653,851	Os06g39810	Os06g0599600	Executor R protein	IRBB27/*Oryza minuta* IRGC101141	Dominant	Gu et al., 2005
*Xa7* ^(a)^	6	28,015,259	–	–	Executor R protein	IRBB7	Dominant	Chen et al., 2021; Wang et al., 2021
*xa13*/*OsSWEET11*/*Os8N3*	8	26,725,952	Os08g42350	Os08g0535200	SWEET-type protein	IRBB13	Recessive	Chu et al., 2006
*xa41(t)*/*OsSWEET14*/*Os11N3*	11	18,171,707	Os11g31190	Os11g0508600	SWEET-type protein	African wild and cultivated rice species *Oryza barthii* and *Oryza glaberrima*	Recessive	Hutin et al., 2015
*Xa21*	11	21,277,443	Os11g36180	Os11g0569733	LRR receptor kinase-like protein	IRBB21 (*Oryza longistaminata* IL)	Dominant	Song et al., 1995
*Xa10*	11	22,181,556	Os11g37570	Os11g0586400	Executor R protein	IRBB10, CAS209	Dominant	Tian et al., 2014
*Xa23*	11	22,204,131	–	Os11g0586701	Executor R protein	CBB23/*Oryza rufipogon*	Dominant	Wang C, et al., 2015
*Xa47(t)*	11	27,983,597	Os11g46200	Os11g0688832	NBS-LRR	*O. rufipogon*	Dominant	Xing et al., 2021
*Xa4* ^(b)^	11	28,357,055	Os11g47140	Os11g0694100	cell wall-associated kinase (WAK)	IRBB4	Dominant	Hu et al., 2017
*Xa3*=*Xa26*	11	28,399,360	Os11g47210	–	LRR receptor kinase-like protein	Minghui 63, IRBB3	Dominant	Sun et al., 2004
*xa25*/*OsSWEET13*/*OsMtN3*	12	17,302,127	Os12g29220	Os12g0476200	SWEET-type protein	Minghui 63	Recessive	Liu et al., 2011

^(a)^The sequence of Xa7 is completely absent in the Nipponbare reference genome (IRGSP1.0) and also most of japonica varieties. Thus, the location of the closest marker (M10) to Xa7 by Chen et al. (2021) is given in the above table.

^(b)^ The sequence of xa4 gene was not fully aligned in the Nipponbare reference genome (IRGSP1.0). Thus, the information of the highest homology sequence was described above.

### 5.2 Bacterial leaf streak (pathogen: *X. oryzae* pv. *oryzicola* (*Xoc*))

For BLS resistance, a handful of QTLs with low-to-moderate PVEs (2.64%–15.93%) were identified (Tang et al., 2000). In addition, a recent GWAS using 510 diverse rice accessions identified 79 quantitative trait nucleotides (QTNs) reflecting 69 QTLs for BLS resistance (Xie et al., 2021). However, no BLS resistance gene has been cloned yet. Among the BLS-resistant QTLs, the highest effect QTL, *qBlsr5a* (12.84%–15.93% PVE), was fine-mapped to 30.0-kb interval on Chr 5, and the resistant parent allele of *Os05g01710* gene within the fine-mapped region was identical to *xa5*, which is one of major BB resistance genes, suggesting that *Os05g01710* (*xa5*) is possibly the candidate gene of *qBlsr5a* (Xie et al., 2014).

### 5.3 Bacterial sheath brown rot also called rice sheath rot (pathogen: *P. fuscovaginae*)

“Rice sheath rot” disease symptoms can be caused by the bacterial pathogen “*P. fuscovaginae*” and also by the fungal pathogen “*S. oryzae*”. A recent pathobiomes study revealed that *P. fuscovaginae* and *S. oryzae* were prevalent in symptomatic rice samples in highland and lowland, respectively, in Burundi, indicating that the pathogens exist independently and are not part of a complex disease (Musonerimana et al., 2020). However, no reliable resistant germplasm and genetic factors have been identified yet.

### 5.4 Bacterial panicle blight, bacterial seedling rot, and bacterial grain rot (pathogen: *B. glumae*)

Bacterial panicle blight (BPB), BSR, and BGR are caused by the same bacterial pathogen, *B. glumae*. It was first reported as BGR in Japan in 1955. Since then, BPB has been found in more than 18 countries globally including Asia, Africa, and North and South America (Zhou, 2019; Ortega and Rojas, 2021). Although it is an emerging disease globally, only several cultivars with partial resistance and 12 QTLs associated with partial resistance have been reported (Zhou, 2019). Regarding BSR resistance, one QTL (*RBG1*/*qRBS1*) was identified from the CSSL population (Nona Bokra introgressions in Koshihikari background) (Mizobuchi et al., 2016). For BGR resistance, 13 QTLs have been found from the two mapping populations: a BIL from Kele (R) × Hitomebore (S) and a RIL from TeQing (R) × Lemont (S) (Mizobuchi et al., 2016).

## 6 Viral diseases and available genetic resources

Seventeen rice viruses have been reported, including rice black-streaked dwarf virus (RBSDV), rice bunchy stunt virus (RBSV), rice dwarf virus (RDV), rice gall dwarf virus (RGDV), rice giallume virus (RGV), RGSV, rice hoja blanca virus (RHBV), rice necrosis mosaic virus (RNMV), RRSV, rice stripe necrosis virus (RSNV), rice stripe virus (RSV), rice transitory yellowing virus (RTYV) also named as rice yellow stunt virus (RYSV), rice tungro bacilliform virus (RTBV), rice tungro spherical virus (RTSV), RYMV, southern rice black-streaked dwarf virus (SRBSDV), and rice stripe mosaic virus (RSMV) (Hibino, 1996; Qin et al., 2019). Since most of the above viruses are arthropod-borne, damages may become more severe as the population of vector insects increases. Among the rice virus diseases, rice tungro disease (RTSV and RTBV), RYMV, and RSV have been causing serious yield loss in South/Southeast Asia, Africa, and temperate Asia, respectively. Thus, a few genes providing resistance to the major viruses above have been cloned. The use of viral disease resistance may significantly reduce the damage of viral diseases. In addition to this, the management of corresponding vector insects may mitigate the damage of viral diseases in the field.

### 6.1 Rice tungro disease caused by RTSV and RTBV

Rice tungro disease is a serious threat to rice production in South and Southeast Asia. Tungro disease viruses are transmitted from tungro-infected plant to another by leafhoppers. The most efficient vector is the green leafhopper (IRRI Rice Knowledge Bank). Tungro was found to be associated with two distinct viruses: RTSV and RTBV. A series of large-scale outbreaks of tungro were recorded in India, Thailand, Indonesia, Malaysia, the Philippines, Thailand, China, and Bangladesh. Tungro, as one of the destructive diseases of rice, causes yield losses of 5% to 10% annually and is estimated to cause an annual loss in rice production of approximately 1.5 billion US dollars worldwide (Dai and Beachy, 2009). In the late 1990s, several tungro-resistant sources, including landrace and wild species, were isolated and used in the breeding program by IRRI, and the most promising breeding lines were developed by crossing with Utri Merah donor (Azzam and Chancellor, 2002). Afterward, Encabo et al. (2009) revealed that RTBV and RTSV are inherited separately from rice accession Utri Merah, conferring resistance to both RTBV and RTSV, and Lee et al. (2010) cloned the causal recessive gene (named as *tsv1*) involved in RTSV resistance in Utri Merah. *TSV1* encodes eukaryotic translation initiation factor 4G (eIF4G), and mutation on the protein-coding sequence of *TSV1* in Utri Merah (*tsv1* allele) may impair the RTSV RNA translation, resulting in tungro resistance. The *tsv1*-Utri Merah allele is widely used for tungro resistance improvement in many breeding programs.

### 6.2 Rice yellow mottle virus

Since RYMV was first discovered in Kenya in 1970, it has been reported from only the countries in the African continent. RYMV causes the most serious damage in Africa among all the rice diseases. Primary infection of RYMV in rice fields is mediated by beetle family chrysomelids, and secondary spread occurs mainly through mechanical contact between infected and healthy leaves by wind (Kouassi et al., 2005). In the past, farmers have been advised to use chemicals to eliminate beetle vectors. The most effective and sustainable way to manage RYMV is to use tolerant and resistant varieties (Abo et al., 1997).

High RYMV resistance was found in one African rice cultivar (*Oryza glaberrima*), Tog5681, and one *O. sativa* cultivar, Gigante. Evaluation of the crosses of these two highly RYMV-resistant cultivars suggests the presence of a single recessive gene (Ndjiondjop et al., 1999). Later, it was discovered that the gene is *RYMV1*, and the gene encodes a eukaryotic translation initiation factor, eIF4(iso)4G (Albar et al., 2006). In sequence comparisons with the dominant susceptible allele (*Rymv1-1*), four different recessive resistant alleles from one *O. sativa* var. Gigante (*rymv1-2*) and three *O. glaberrima* accessions (Tog5681 (*rymv1-3*), Tog5672 (*rymv1-4*), and Tog5674 (*rymv1-5*)) were characterized by the presence of short amino acid substitutions or short deletions in the MIF4G domain of the protein (Albar et al., 2006; Thiémélé et al., 2010). Allele-specific markers targeting mutations or deletions characterizing different *RYMV1* were developed for improving MAS for the introduction of the resistance alleles into susceptible cultivars of *O. sativa* or *O. glaberrima* (Thiémélé et al., 2010). In the second major recessive resistance gene, *RYMV2*, it was identified that 1-bp deletion on the coding sequence of the rice homolog of the *Arabidopsis CPR5* gene, known to be a defense mechanism regulator, from the resistant African rice (*O. glaberrima*) Tog7291 provided RYMV resistance (Orjuela et al., 2013). A single dominant resistant gene *RYMV3* encoding NBS-LRR protein was identified from the *O. glaberrima* Tog5307 (Pidon et al., 2017). Novel resistant alleles and accessions for *RYMV2* and *RYMV3* were identified by screening 268 *O. glaberrima* accessions and sequencing (Pidon et al., 2020), and five new resistant germplasm were isolated from Korean rice lines (Asante et al., 2020). The cloned genes with different resistant alleles will be useful to improve RYMV resistance, especially for the breeding program for the African continent.

### 6.3 Rice stripe virus

RSV is an RNA-type virus belonging to the genus *Tenuivirus*, and it is transmitted by SBPHs. RSV has been reported only in China, Japan, Korea, and Taiwan, where *japonica* rice is cultivated, and it caused severe damage to the rice fields in Eastern China, Japan, and Korea. While most *indica* varieties are resistant to RSV, the majority of *japonica* varieties are highly susceptible. A number of RSV-resistant QTLs have been reported from diverse *indica*-resistant donors, and the major QTLs were repeatedly detected on Chr 11 among several QTL mapping (Cho et al., 2013). Finally, the major QTL, *qSTV11*, originated from an *indica* variety Kasalath and was cloned (Wang et al., 2014). *STV11*-Kasalath allele encodes a sulfotransferase (OsSOT1) protein catalyzing the conversion of salicylic acid (SA) into sulfonated SA (SSA), whereas the protein encoded by the susceptible allele *STV11* loses this activity. *STV11* gene will be useful in improving RSV resistance in the *japonica* varieties.

## 7 Physical locations of the cloned genes/alleles on rice chromosomes

Graphical mapping of the cloned genes on 12 rice chromosomes will be useful information for MAS breeding, especially for gene pyramiding, as well as mapping new biotic stress resistance genes. We mapped the physical locations of all the cloned 48 biotic stress resistance genes on the 12 rice chromosomes (**Figure 1**). The cloned genes were not evenly distributed across the rice genome. No biotic stress resistance gene was cloned yet on Chr 10. In contrast, Chr 11 possesses the highest number of genes (15 genes), following Chr 6 (eight genes), Chr 4 (seven genes), and Chr 12 (four genes), with these four chromosomes harboring 34 genes out of 48 cloned genes (70.83%). Interestingly, 14 cloned genes associated with blast, bacterial blight, and virus resistance were on the 10.41-Mb region of the long arm of Chr 11 (Chr 11: 17.98–28.39 Mb), and it took 29.16% of the cloned genes. Biotic stress resistance genes are ~10 times more enriched in this specific region than any other loci (the expected distribution is ~1.2 cloned gene/10 Mb). Another interesting point is that the bacterial blight resistance gene *Xa47(t)* (*Os11g46200*) encoding NBS-LRR is overlapped with the blast resistance gene *Pik*/*Pik-m*/*Pik-p*/*Pi1*/*Pi7* consisting of two NBS-LRR genes (*Os11g46200* and *Os11g46210*). In some loci, different resistance alleles at the same locus, such as *BPH1* locus, *Pi9* locus, *Pik* locus, and *Xa1* locus, were identified (**Tables 1**–**3**). Although some of them among the alleles showed different reactions to pathotypes, unfortunately, they cannot be pyramided by MAS due to the same physical location among the alleles. Thus, breeders need to choose one suitable allele among the alleles based on the regional pathotypes/isolates. Similarly, in gene pyramiding/stacking, breeders should also consider the physical distance between/among the target genes. If the two target genes are closely located with each other (<~1Mb) on the same chromosome (for example, *Xa10* and *Pb1* on Chr 11, *Pik* and *Xa4* on Chr 11, and *Pita* and *Ptr*=*Pita2* on Chr 12; see **Figure 1**), breeders need to produce many progenies to obtain the gene pyramided plants through the selection of the recombinant plants between the two target loci. In rice, a handful of recombination hot and cold regions are reported, and the average recombination frequency is approximately 4.35 cM per Mb (Si et al., 2015). In addition, breeders also need to check the target loci whether the important genes governing other agronomic traits are present near the target biotic stress resistance gene to avoid linkage drag. For instance, a key amylose synthesis gene *Waxy*/*GBSS1* (1.76-Mb location on Chr 6) is tightly linked with *BPH32* (1.22 Mb on Chr 6), and a major heading date gene *Hd1* (9.33 Mb on Chr 6) is closely located with *Pi2* gene (10.38 Mb on Chr 6). Thus, breeders should consider the locations of the important agronomic traits genes near the target genes, especially when the breeders try to retain the original characteristics of the elite background variety, except for the target biotic stress resistance. A map of the physical locations of the cloned genes (**Figure 1**) will be helpful for consideration of the above points in MAS breeding programs.

## 8 Available DNA markers for MAS breeding

DNA markers are essential tools for genetic analysis as well as marker-assisted breeding. We tried to collect all the markers published and used in the previous breeding programs, and we collected ~500 markers in total for the cloned biotic stress resistance genes (**Table S1**). We filed essential information on the markers for the potential users, including marker types (InDel, CAPS/dCAPS, dominant markers, and tetra-primer method markers) and primer sequences. Also, we cited the original references of each marker so that breeders can obtain detailed and additional information if needed. Furthermore, we mapped the location of all the markers in the rice reference genome sequence (Os-Nipponbare-Reference-IRGSP-1.0: https://rapdb.dna.affrc.go.jp/). This information provides physical distance between the target gene and the markers, and it will be helpful to reduce the selection of false positives during MAS. For examples, some markers for the *BPH1*, *BP17*, *xa13*, *Xa27*, *Pi9*, *Piz-t*, *Pizh*, *Pish*, *Pi5*, *Pita2*, and *RYMV1* genes/alleles are a bit far (>1 Mb) from the gene locus (**Table S1**). Selection of genic or gene-tightly linked markers would reduce false-positive selection. In cases of multi-alleles for the same gene, such as *BPH1* and *Pi9*, all the available markers for the same gene can be tested to check the possibility of polymorphism between the parents, and the selected polymorphic markers can be used in MAS breeding (for example, *BPH18* markers for *BPH26* MAS breeding). All the information on the markers is described in **Table S1**.

## 9 Conclusions and perspective

In this review, we summarized all the cloned genes associated with biotic stress resistance (**Tables 1**–**4**), mapped the physical location of the genes on 12 rice chromosomes (**Figure 1**), and consolidated the available markers associated with the cloned genes (**Table S1**). Furthermore, we also briefly introduced genetic resources such as QTLs and donor sources for some biotic stress if the cloned genes are not available yet. The information presented in this review will be helpful for checking the available genetic resources for biotic stress resistance and also for MAS breeding for the genetic improvement of biotic stress resistance in rice. As shown in many previous reports, pyramiding of QTLs/genes might be a practical solution to breed durable and broad-spectrum resistant varieties.

**Table 4:** The cloned virus resistance genes.

Gene	Chr	Location (bp)	MSU_ID	RAPDB_ID	Encoding protein	Resistant/donor allele	Inheritance pattern of R-allele	Reference
*tsv1*	7	22,114,961	Os07g36940	Os07g0555200	Eukaryotic translation initiation factor 4G (eIF4G)	Utri Merah (UM82)	Recessive	Lee et al., 2010
*RYMV1*	4	24,946,171	Os04g42140	Os04g0499300	Eukaryotic translation initiation factor isoform 4G-1 (eIF(iso)4G1)	*Oryza sativa* Gigante (*rymv1-2*)/*Oryza glaberrima* accessions Tog5681, Tog5672, and Tog5674 for *rymv1-3*, *rymv-4*, and *rymv-5*, respectively	Recessive	Albar et al., 2006; Thiémélé et al., 2010
*RYMV2*	1	40,073,727	Os01g68970	Os01g0918500	Constitutive expresser of PR genes5 (CPR5)	*O. glaberrima* Tog7291	Recessive	Orjuela et al., 2013
*RYMV3*	11	26,380,866	Os11g43700	Os11g0657900	NBS-LRR	*O. glaberrima* Tog5307	Dominant	Pidon et al., 2017
*STV11*	11	17,985,011	Os11g30910	Os11g0505300	Sulfotransferase (OsSOT1)	Kasalath	ND	Wang et al., 2014

ND, not determined.

Approximately 48 genes, which are natural alleles and provide biotic stress resistance, have been cloned only for the major biotic stresses, including BPH, blast, BB, and some viruses. However, no genes have been cloned yet for other biotic stresses. Preparation of the reliable genetic factors (genes/QTLs) associated with currently problematic and emerging pathogens is very important for stable high-yield rice production, and thus, scientists/geneticists need to put much effort into this pending issue. Screening wild relatives of rice in the genus *Oryza* will be one of the ideal approaches. Many biotic stress resistance genes were already cloned from wild germplasm (see **Tables 1**–**3**), such as *BPH14* (*O. officinalis*), *Pi9* (*Oryza minuta*), and *Xa21* (*O. longistaminata*). More than 4,500 accessions of wild rice species are stored in the IRRI Genebank (Banaticla-Hilario and Sajise, 2022), and most of the germplasms were not screened yet. Recently, a genome-wide InDel marker set (475 polymorphic markers) discriminating the alleles between *O. sativa* and the other seven AA-genome *Oryza* species was developed to harness AA-genome wild species (Hechanova et al., 2021). The genes identified from wild germplasm will be rare alleles due to mostly untapped and unused materials in breeding, and thus, they will be effective in most *indica* and *japonica* backgrounds.

The incidence of pathogens and insect pests will change in time and space; notably, it will be also influenced by climate changes. As examples, some BPH resistance genes were affected by artificial climate change conditions (the atmospheric temperature with corresponding carbon dioxide at the ambient, year 2050 and year 2100) (Kuang et al., 2021) and also by nitrogen fertilizer treatments (Lin et al., 2022). Moreover, most of the genes/QTLs reported were tested with limited numbers of isolates/biotypes, which were collected in specific locations and years. Thus, the identified genes/QTLs could not guarantee resistance across locations, time, and environments. Testing donor germplasm, especially sets of NILs possessing specific genes/QTLs such as NILs for BPH (Jena et al., 2017), blast (Telebanco-Yanoria et al., 2010; Telebanco-Yanoria et al., 2011; Fukuta et al., 2022), and BB (Ogawa et al., 1991; IRBB lines), with prevalence races/biotypes in the target regions, would be a good strategy to select effective genes/alleles in breeding program.

DNA markers are essential tools for genetic analysis and breeding. DNA markers could reduce the time and effort in developing and improving biotic-resistant cultivars through marker-assisted breeding. Due to the accessibility and technical simplicity for the rice breeders, most of the markers are PCR and gel-based markers, including SSR (RM) markers, InDel markers, CAPS markers, tetra-primer PCR markers, and dominant PCR markers (**Table S1**). These markers have contributed much to MAS breeding. However, the gene/allele-specific markers are limited to some specific genes, and a high portion of the markers are the gene-linked makers (sometimes more than a few Mb distance from the gene), probably causing that false-positive selection in MAS breeding. Thus, breeders should check the marker–gene linkage (distance between the gene and markers) and also marker quality (reproducibility and polymorphism between parents) before starting MAS breeding. For efficient and precious introgression of the target genes, currently, available markers might be insufficient. Developments of breeder-friendly allele-specific markers and enough number of polymorphic markers with high reproducibility for many biotic stress resistance genes/alleles are urgently needed. This will help the rapid deployment of target biotic stress resistance genes in the elite local varieties.

In addition to MAS breeding, CRISPR-based genome editing technologies might be an alternative solution for the fast improvement of biotic stress resistance. The advantage of genome editing is that the techniques can directly improve target traits in elite backgrounds without crossing with the donor lines. Thus, some unexpected phenotypes caused by linkage drag or other donor introgressions happening during MAS breeding will not be considered in genome editing-based trait improvement. Recently, its potential was already shown in BB resistance improvement by CRISPR-based promoter editing of three *SWEET* genes in rice (Oliva et al., 2019) and in tungro virus resistance by editing of *TSV1* gene (Macovei et al., 2018). Another advantage is that genome-edited products are regulated with lesser stringency in many countries compared to conventional genetically modified organisms (GMOs). Together with cross-based breeding, genome editing technologies can contribute fast genetic improvement of target traits in the elite variety backgrounds without linkage drag and other donor introgressions.

## Author contributions

JH, C-PL, AT, E-KA, JJ, I-RC, RS, KJ, and S-RK conceived this review paper. ES, SH, I-RC, and S-RK performed the literature search and wrote the draft. The manuscript was improved by revisions by all the authors. All authors agreed to the published version of the manuscript.

## References

[B1] Abo M. E. Sy A. A. Alegbejo M. D. (1997). Rice yellow mottle virus (RYMV) in Africa: evolution, distribution, economic significance on sustainable rice production and management strategies. J. Sustain. Agric. 11 (2-3), 85–111. doi: 10.1300/J064v11n02_08

[B2] Addison C. K. Angira B. Cerioli T. Groth D. E. Richards J. K. Linscombe S. D.. (2021). Identification and mapping of a novel resistance gene to the rice pathogen, *Cercospora janseana* . Theor. Appl. Genet. 134, 2221–2234. doi: 10.1007/s00122-021-03821-2 33825949

[B3] Albar L. Bangratz-Reyser M. Hébrard E. Ndjiondjop M. N. Jones M. Ghesquière A. (2006). Mutations in the eIF (iso) 4G translation initiation factor confer high resistance of rice to rice yellow mottle virus. Plant J. 47 (3), 417–426. doi: 10.1111/j.1365-313X.2006.02792.x 16774645

[B4] Andargie M. Li L. Feng A. Zhu X. Li J. (2018). Mapping of the quantitative trait locus (QTL) conferring resistance to rice false smut disease. Curr. Plant Biol. 15, 38–43. doi: 10.1016/j.cpb.2018.11.003

[B5] Asante M. D. Amadu B. Traore V. S. E. Oppong A. Adebayo M. A. Aculey P.. (2020). Assessment of Korean rice lines for their reaction to rice yellow mottle virus in Ghana. Heliyon 6 (11), e05551. doi: 10.1016/j.heliyon.2020.e05551 33294693 PMC7691548

[B6] Ashikawa I. Hayashi N. Yamane H. Kanamori H. Wu J. Matsumoto T.. (2008). Two adjacent nucleotide-binding site–leucine-rich repeat class genes are required to confer Pikm-specific rice blast resistance. Genetics 180 (4), 2267–2276. doi: 10.1534/genetics.108.095034 18940787 PMC2600957

[B7] Ashkani S. Rafii M. Y. Shabanimofrad M. Ghasemzadeh A. Ravanfar S. A. Latif M. A. (2016). Molecular progress on the mapping and cloning of functional genes for blast disease in rice (*Oryza sativa* L.): current status and future considerations. Crit. Rev. Biotechnol. 36 (2), 353–367. doi: 10.3109/07388551.2014.961403 25394538

[B8] Azzam O. Chancellor T. C. (2002). The biology, epidemiology, and management of rice tungro disease in Asia. Plant Dis. 86 (2), 88–100. doi: 10.1094/PDIS.2002.86.2.88 30823328

[B9] BalachIranjeevi C. Naik B. Kumar A. Harika G. Hajira S. Kumar D. (2018). Marker-assisted pyramiding of two major broad-spectrum bacterial blight resistance genes, *Xa21* and *Xa33* into an elite maintainer line of rice, DRR17B. PLoS One 13 (10), e0201271. doi: 10.1371/journal.pone.0201271 30359375 PMC6201878

[B10] Banaticla-Hilario M. C. N. Sajise A. G. (2022). “Recent developments in wild rice conservation, research, and use”. in Plant Genet. Resources Inventory Collection Conserv. eds. Ramamoorthy S. Buot I.J. Chandrasekaran R. (Singapore: Springer), 43–76. doi: 10.1007/978-981-16-7699-4_3

[B11] Bandumula N. (2018). Rice production in Asia: key to global food security. Proc.Natl. Acad. Sci. 88, 1323–1328. doi: 10.1007/s40011-017-0867-7

[B12] Bebber D. P. (2015). Range-expanding pests and pathogens in a warming world. Annu. Rev. Phytopathol. 53, 335–356. doi: 10.1146/annurev-phyto-080614-120207 26047565

[B13] Bentur J. S. Rawat N. Divya D. Sinha D. K. Agarrwal R. Atray I.. (2016). Rice–gall midge interactions: battle for survival. J. Insect Physiol. 84, 40–49. doi: 10.1016/j.jinsphys.2015.09.008 26455891

[B14] Beser N. Del Valle M. M. Kim S. M. Vinarao B. R. Surek H. Jena K. K. (2016). Marker-assisted introgression of a broad-spectrum resistance gene, Pi40 improved blast resistance of two elite rice (*Oryza sativa* L.). Mol. Plant Breed. 7, 1–15. doi: 10.5376/mpb.2016.07.0033

[B15] Bigirimana V. D. P. Hua G. K. Nyamangyoku O. I. Höfte M. (2015). Rice sheath rot: an emerging ubiquitous destructive disease complex. Front. Plant Sci. 6 1066. doi: 10.3389/fpls.2015.01066 26697031 PMC4675855

[B16] Blair M. W. Garris A. J. Iyer A. S. Chapman B. Kresovich S. McCouch S. R. (2003). High resolution genetic mapping and candidate gene identification at the *xa5* locus for bacterial blight resistance in rice (*Oryza sativa* L.). Theoret. Appl. Genet. 107, 62–73. doi: 10.1007/s00122-003-1231-2 12677405

[B17] Brown J. K. (2002). Yield penalties of disease resistance in crops. Curr. Opin. Plant Biol. 5 (4), 339–344. doi: 10.1016/S1369-5266(02)00270-4 12179968

[B18] Bryan G. T. Wu K. S. Farrall L. Jia Y. Hershey H. P. McAdams S. A.. (2000). A single amino acid difference distinguishes resistant and susceptible alleles of the rice blast resistance gene *Pi-ta* . Plant Cell 12 (11), 2033–2045. doi: 10.2307/3871103 11090207 PMC150156

[B19] Cabauatan P. Q. Cabunagan R. C. Choi I. R. (2009). “Rice viruses transmitted by the brown planthopper *Nilaparvata lugens* Stål.”, in Planthoppers: New threats to sustainability Intensive Rice production Syst. Asia eds. Heong K.L. Hardy B. (Philippines: International Rice Research Institute), 357–368.

[B20] Cesari S. Thilliez G. Ribot C. Chalvon V. Michel C. Jauneau A.. (2013). The rice resistance protein pair RGA4/RGA5 recognizes the *Magnaporthe oryzae* effectors AVR-Pia and AVR1-CO39 by direct binding. Plant Cell 25 (4), 1463–1481. doi: 10.1105/tpc.112.107201 23548743 PMC3663280

[B21] Chaloner T. M. Gurr S. J. Bebber D. P. (2021). Plant pathogen infection risk tracks global crop yields under climate change. Nat. Clim. Change 11 (8), 710–715. doi: 10.1038/s41558-021-01104-8

[B22] Chen H. He H. Zou Y. Chen W. Yu R. Liu X.. (2011). Development and application of a set of breeder-friendly SNP markers for genetic analyses and molecular breeding of rice (Oryza sativa L.). Theor. Appl. Genet. 123, 869–879. doi: 10.1007/s00122-011-1633-5 21681488

[B23] Chen X. Liu P. Mei L. He X. Chen L. Liu H.. (2021). *Xa7*, a new executor R gene that confers durable and broad-spectrum resistance to bacterial blight disease in rice. Plant Com 2 (3), 100143. doi: 10.1016/j.xplc.2021.100143 PMC8132130 34027390

[B24] Chen X. Shang J. Chen D. Lei C. Zou Y. Zhai W.. (2006). AB-lectin receptor kinase gene conferring rice blast resistance. Plant J. 46 (5), 794–804. doi: 10.1111/j.1365-313X.2006.02739.x 16709195

[B25] Chen J. Shi Y. Liu W. Chai R. Fu Y. Zhuang J.. (2011). A Pid3 allele from rice cultivar Gumei2 confers resistance to Magnaporthe oryzae. J. Genet. Genomics 38 (5), 209–216. doi: 10.1016/j.jgg.2011.03.010 21621742

[B26] Chen Z. Zhao W. Zhu X. Zou C. Yin J. Chern M.. (2018). Identification and characterization of rice blast resistance gene *Pid4* by a combination of transcriptomic profiling and genome analysis. J. Genet. Genomics 45 (12), 663–672. doi: 10.1016/j.jgg.2018.10.007 30606471

[B27] Cho W. K. Lian S. Kim S. M. Park S. H. Kim K. H. (2013). Current insights into research on Rice stripe virus. Plant Pathol. J. 29 (3), 223. doi: 10.5423/PPJ.RW.10.2012.0158 25288949 PMC4174810

[B28] Chu Z. Fu B. Yang H. Xu C. Li Z. Sanchez A.. (2006). Targeting *xa13*, a recessive gene for bacterial blight resistance in rice. Theoret. Appl. Genet. 112, 455–461. doi: 10.1007/s00122-005-0145-6 16328230

[B29] Dai S. Beachy R. N. (2009). Genetic engineering of rice to resist rice tungro disease. In Vitro Cell. Dev. Biol. - Plant 45, 517–524. doi: 10.1007/s11627-009-9241-7

[B30] Das A. Soubam D. Singh P. K. Thakur S. Singh N. K. Sharma T. R. (2012). A novel blast resistance gene, *Pi54rh* cloned from wild species of rice, *Oryza rhizomatis* confers broad spectrum resistance to Magnaporthe oryzae. Funct. Integrat. Genom. 12, 215–228. doi: 10.1007/s10142-012-0284-1 22592658

[B31] Deng Y. Zhai K. Xie Z. Yang D. Zhu X. Liu J.. (2017). Epigenetic regulation of antagonistic receptors confers rice blast resistance with yield balance. Science 355 (6328), 962–965. doi: 10.1126/science.aai8898 28154240

[B32] Divya D. Himabindu K. Nair S. Bentur J. S. (2015). Cloning of a gene encoding LRR protein and its validation as candidate gall midge resistance gene, *Gm4*, in rice. Euphytica 203, 185–195. doi: 10.1007/s10681-014-1302-2

[B33] Divya D. Sahu N. Nair S. Bentur J. S. (2018). Map-based cloning and validation of a gall midge resistance gene, *Gm8*, encoding a proline-rich protein in the rice variety Aganni. Mol. Biol. Rep. 45 (6), 2075–2086. doi: 10.1007/s11033-018-4364-8 30209741

[B34] Dixit S. Singh U. M. Singh A. K. Alam S. Venkateshwarlu C. Nachimuthu V. V.. (2020). Marker assisted forward breeding to combine multiple biotic-abiotic stress resistance/tolerance in rice. Rice 13, 1–15. doi: 10.1186/s12284-020-00391-7 32472217 PMC7260318

[B35] Du B. Chen R. Guo J. He G. (2020). Current understanding of the genomic, genetic, and molecular control of insect resistance in rice. Mol. Breed. 40, 1–25. doi: 10.1007/s11032-020-1103-3

[B36] Du B. Zhang W. Liu B. Hu J. Wei Z. Shi Z.. (2009). Identification and characterization of *Bph14*, a gene conferring resistance to brown planthopper in rice. Proc. Natl. Acad. Sci. 106 (52), 22163–22168. doi: 10.1073/pnas.0912139106 20018701 PMC2793316

[B37] Dyck V. A. Thomas B. (1979). “The brown planthopper problem,” in Brown planthopper: threat to rice production in Asia (Los Baños, Philippines: International Rice Research Institute), 3–17.

[B38] Encabo J. R. Cabauatan P. Q. Cabunagan R. C. Satoh K. Lee J. H. Kwak D. Y.. (2009). Suppression of two tungro viruses in rice by separable traits originating from cultivar Utri Merah. Mol. Plant Microbe Interact. 22 (10), 1268–1281. doi: 10.1094/MPMI-22-10-1268 19737100

[B39] Fahad S. Adnan M. Noor M. Arif M. Alam M. Khan I. A.. (2019). “Major constraints for global rice production,” in Advances in rice research for abiotic stress tolerance. Eds. Hasanuzzaman M. Fujita M. Nahar K. Biswas J. K. (United Kingdom: Woodhead Publishing), 1–22.

[B40] Figoni R. A. Rutger J. N. Webster R. K. (1983). Evaluation of wild *Oryza* species for stem rot (*Sclerotium oryzae*) resistance. Plant Dis. 67 (9), 998–1000. doi: 10.1094/PD-67-998

[B41] Fisher M. C. Henk D. A. Briggs C. J. Brownstein J. S. Madoff L. C. McCraw S. L.. (2012). Emerging fungal threats to animal, plant and ecosystem health. Nature 484 (7393), 186–194. doi: 10.1038/nature10947 22498624 PMC3821985

[B42] Fujita D. Kohli A. Horgan F. G. (2013). Rice resistance to planthoppers and leafhoppers. Crit. Rev. Plant Sci. 32, 162–191. doi: 10.1080/07352689.2012.735986

[B43] Fukuoka S. Saka N. Koga H. Ono K. Shimizu T. Ebana K.. (2009). Loss of function of a proline-containing protein confers durable disease resistance in rice. Science 325 (5943), 998–1001. doi: 10.1126/science.117555 19696351

[B44] Fukuoka S. Yamamoto S. I. Mizobuchi R. Yamanouchi U. Ono K. Kitazawa N.. (2014). Multiple functional polymorphisms in a single disease resistance gene in rice enhance durable resistance to blast. Sci. Rep. 4 (1), 1–7. doi: 10.1038/srep04550

[B45] Fukuta Y. Koide Y. Kobayashi N. Kato H. Saito H. Telebanco-Yanoria M. J.. (2022). Lines for blast resistance genes with genetic background of Indica Group rice as international differential variety set. Plant Breed. 141 (5), 609–620. doi: 10.1111/pbr.13040

[B46] Gan L. Zhai C. Hua L. (2010). Rice blast resistance gene Pi7 and application thereof. CN patent Application no: CN102094027A (South China Agricultural University, China).

[B47] Goad D. M. Jia Y. Gibbons A. Liu Y. Gealy D. Caicedo A. L.. (2020). Identification of novel QTL conferring sheath blight resistance in two weedy rice mapping populations. Rice 13, 1–10. doi: 10.1186/s12284-020-00381-9 32206941 PMC7090113

[B48] Gu K. Yang B. Tian D. Wu L. Wang D. Sreekala C.. (2005). R gene expression induced by a type-III effector triggers disease resistance in rice. Nature 435 (7045), 1122–1125. doi: 10.1038/nature03630 15973413

[B49] Guo J. Xu C. Wu D. Zhao Y. Qiu Y. Wang X.. (2018). *Bph6* encodes an exocyst-localized protein and confers broad resistance to planthoppers in rice. Nat. Genet. 50 (2), 297–306. doi: 10.1038/s41588-018-0039-6 29358653

[B50] Han Y. Li D. Yang J. Huang F. Sheng H. Sun W. (2020). Mapping quantitative trait loci for disease resistance to false smut of rice. Phytopathol. Res. 2, 1–11. doi: 10.1186/s42483-020-00059-6

[B51] Hayashi N. Inoue H. Kato T. Funao T. Shirota M. Shimizu T.. (2010). Durable panicle blast-resistance gene *Pb1* encodes an atypical CC-NBS-LRR protein and was generated by acquiring a promoter through local genome duplication. Plant J. 64 (3), 498–510. doi: 10.1111/j.1365-313X.2010.04348.x 20807214

[B52] Hayashi K. Yasuda N. Fujita Y. Koizumi S. Yoshida H. (2010). Identification of the blast resistance gene *Pit* in rice cultivars using functional markers. Theoret. Appl. Genet. 121, 357–1367. doi: 10.1007/s00122-010-1393-7 20589366

[B53] Hechanova S. L. Bhattarai K. Simon E. V. Clave G. Karunarathne P. Ahn E. K.. (2021). Development of a genome-wide InDel marker set for allele discrimination between rice (*Oryza sativa*) and the other seven AA-genome *Oryza* species. Sci. Rep. 11 (1), 8962. doi: 10.1038/s41598-021-88533-9 33903715 PMC8076200

[B54] Hibino H. (1996). Biology and epidemiology of rice viruses. Annu. Rev. Phytopathol. 34, 249–274. doi: 10.1146/annurev.phyto.34.1.249 15012543

[B55] Hiremath S. S. Bhatia D. Jain J. Hunjan M. S. Kaur R. Zaidi N. W.. (2021). Identification of potential donors and QTLs for resistance to false smut in a subset of rice diversity panel. Eur. J. Plant Pathol. 159, 461–470. doi: 10.1007/s10658-020-02172-w

[B56] Horgan F. G. Almazan M. L. P. Vu Q. Ramal A. F. Bernal C. C. Yasui H.. (2019). Unanticipated benefits and potential ecological costs associated with pyramiding leafhopper resistance loci in rice. Crop Prot. 115, 47–58. doi: 10.1016/j.cropro.2018.09.013 30739972 PMC6358143

[B57] Horgan F. G. Bernal C. C. Vu Q. Almazan M. L. P. Ramal A. F. Yasui H.. (2018). Virulence adaptation in a rice leafhopper: Exposure to ineffective genes compromises pyramided resistance. Crop Prot. 113, 40–47. doi: 10.1016/j.cropro.2018.07.010 30393420 PMC6106693

[B58] Hsu Y. C. Chiu C. H. Yap R. Tseng Y. C. Wu Y. P. (2020). Pyramiding bacterial blight resistance genes in Tainung82 for broad-spectrum resistance using marker-assisted selection. Int. J. Mol. Sci. 21 (4), 1281. doi: 10.3390/ijms21041281 32074964 PMC7072918

[B59] Hu K. Cao J. Zhang J. Xia F. Ke Y. Zhang H.. (2017). Improvement of multiple agronomic traits by a disease resistance gene via cell wall reinforcement. Nat. Plants 3 (3), 1–9. doi: 10.1038/nplants.2017.9 28211849

[B60] Hu J. Li X. Wu C. Yang C. Hua H. Gao G. (2012). Pyramiding and evaluation of the brown planthopper resistance genes *Bph14* and *Bph15* in hybrid rice. Mol. Breed. 29, 61–69. doi: 10.1007/s11032-010-9526-x

[B61] Hu J. Xiao C. He Y. (2016). Recent progress on the genetics and molecular breeding of brown planthopper resistance in rice. Rice 9 (1), 1–12. doi: 10.1186/s12284-016-0099-0 27300326 PMC4908088

[B62] Hua L. Wu J. Chen C. Wu W. He X. Lin F.. (2012). The isolation of *Pi1*, an allele at the *Pik* locus which confers broad spectrum resistance to rice blast. Theoret. Appl. Genet. 125, 1047–1055. doi: 10.1007/s00122-012-1894-7 22643901

[B63] Hutin M. Sabot F. Ghesquière A. Koebnik R. Szurek B. (2015). A knowledge-based molecular screen uncovers a broad-spectrum *Os SWEET 14* resistance allele to bacterial blight from wild rice. Plant J. 84 (4), 694–703. doi: 10.1111/tpj.13042 26426417

[B64] Inoue H. Nakamura M. Mizubayashi T. Takahashi A. Sugano S. Fukuoka S.. (2017). Panicle blast 1 (*Pb1*) resistance is dependent on at least four QTLs in the rice genome. Rice 10 (1), 1–10. doi: 10.1186/s12284-017-0175-0 28766258 PMC5539066

[B65] Inukai T. Nagashima S. Kato M. (2019). *Pid3-I1* is a race-specific partial-resistance allele at the *Pid3* blast resistance locus in rice. Theor. Appl. Genet. 132, 395–404. doi: 10.1007/s00122-018-3227-y 30390130

[B66] IRRI Rice Knowledge Bank. Available at: http://www.knowledgebank.irri.org/step-by-step-production/growth/pests-and-diseases.

[B67] Iyer A. S. McCouch S. R. (2004). The rice bacterial blight resistance gene *xa5* encodes a novel form of disease resistance. Mol. Plant Microbe Interact. 17, 1348–1354. doi: 10.1094/MPMI.2004.17.12.1348 15597740

[B68] Jairin J. Phengrat K. Teangdeerith S. Vanavichit A. Toojinda T. (2007). Mapping of a broad-spectrum brown planthopper resistance gene, *Bph3*, on rice chromosome 6. Mol. Breed. 19, 35–44. doi: 10.1007/s11032-006-9040-3

[B69] Jamaloddin M. Mahender A. Gokulan C. G. BalachIranjeevi C. Maliha A. Patel H. K.. (2021). “Molecular approaches for disease resistance in rice”, Rice Improvement, eds. Ali J. Wani S. H. (Cham: Springer) 315–378. doi: 10.1007/978-3-030-66530-2_10

[B70] Jena K. K. Hechanova S. L. Verdeprado H. Prahalada G. D. Kim S. R. (2017). Development of 25 near-isogenic lines (NILs) with ten BPH resistance genes in rice (*Oryza sativa* L.): production, resistance spectrum, and molecular analysis. Theor. Appl. Genet. 130, 2345–2360. doi: 10.1007/s00122-017-2963-8 28795219

[B71] Jena K. K. Kim S. M. (2010). Current status of brown planthopper (BPH) resistance and genetics. Rice 3, 161–171. doi: 10.1007/s12284-010-9050-y

[B72] Jeung J. U. Heu S. G. Shin M. S. Cruz C. M. Jena K. K. (2006). Dynamics of *Xanthomonas oryzae* pv. *oryzae* populations in Korea and their relationship to known bacterial blight resistance genes. Phytopathology 96, 867–875. doi: 10.1094/PHYTO-96-0867 18943752

[B73] Jeung J. U. Kim B. R. Cho Y. C. Han S. S. Moon H. P. Lee Y. T.. (2007). A novel gene, Pi40 (t), linked to the DNA markers derived from NBS-LRR motifs confers broad spectrum of blast resistance in rice. Theor. Appl. Genet. 115, 1163–1177. doi: 10.1007/s00122-007-0642-x 17909744

[B74] Ji C. Ji Z. Liu B. Cheng H. Liu H. Liu S.. (2020). *Xa1* allelic R genes activate rice blight resistance suppressed by interfering TAL effectors. Plant Commun. 1 (4), 100087. doi: 10.1016/j.xplc.2020.100087 33367250 PMC7748017

[B75] Ji H. Kim S. R. Kim Y. H. Suh J. P. Park H. M. Sreenivasulu N.. (2016). Map-based cloning and characterization of the *BPH18* gene from wild rice conferring resistance to brown planthopper (BPH) insect pest. Sci. Rep. 6 (1), 1–14. doi: 10.1038/srep34376 27682162 PMC5041133

[B76] Jiang H. Li Z. Liu J. Shen Z. Gao G. Zhang Q.. (2019). Development and evaluation of improved lines with broad-spectrum resistance to rice blast using nine resistance genes. Rice 12 (1), 29. doi: 10.1186/s12284-019-0292-z 31062101 PMC6502921

[B77] Jiang N. Yan J. Liang Y. Shi Y. He Z. Wu Y.. (2020). Resistance genes and their interactions with bacterial blight/leaf streak pathogens (*Xanthomonas oryzae*) in rice (*Oryza sativa* L.)—an updated review. Rice 13 (1), 1–12. doi: 10.1186/s12284-019-0358-y 31915945 PMC6949332

[B78] Kabish A. Khush G. S. (1988). Genetic analysis of resistance to brown planthopper in rice (*Oryza sativa* L.). Plant Breed. 100, 54–58. doi: 10.1111/j.1439-0523.1988.tb00216.x

[B79] Ke Y. Deng H. Wang S. (2017). Advances in understanding broad-spectrum resistance to pathogens in rice. Plant J. 90 (4), 738–748. doi: 10.1111/tpj.13438 27888533

[B80] Khush G. S. (2005). What it will take to feed 5.0 billion rice consumers in 2030. Plant Mol. Biol. 59, 1–6. doi: 10.1007/s11103-005-2159-5 16217597

[B81] Kim S. R. Ramos J. Ashikari M. Virk P. S. Torres E. A. Nissila E.. (2016). Development and validation of allele-specific SNP/indel markers for eight yield-enhancing genes using whole-genome sequencing strategy to increase yield potential of rice, *Oryza sativa* L. Rice 9 (1), 1–17. doi: 10.1186/s12284-016-0084-7 26987543 PMC4797370

[B82] Kim E. G. Yun S. Park J. R. Kim K. M. (2021). Identification of F3H, major secondary metabolite-related gene that confers resistance against whitebacked planthopper through QTL mapping in rice. Plants 10 (1), 81. doi: 10.3390/plants10010081 33401742 PMC7823371

[B83] Kouassi N. K. N'guessan P. Albar L. Fauquet C. M. Brugidou C. (2005). Distribution and characterization of Rice yellow mottle virus: a threat to African farmers. Plant Dis. 89 (2), 124–133. doi: 10.1094/PD-89-0124 30795214

[B84] Kuang Y.-H. Fang Y.-F. Lin S.-C. Tsai S.-F. Yang Z.-W. Li C.-P.. (2021). The impact of climate change on the resistance of rice near-isogenic lines with resistance genes against brown planthopper. Rice 14, 64. doi: 10.1186/s12284-021-00508-6 34337676 PMC8326240

[B85] Lanoiselet V. M. Cother E. J. Ash G. J. (2007). Aggregate sheath spot and sheath spot of rice. Crop Prot. 26, 799–808. doi: 10.1016/j.cropro.2006.06.016

[B86] Lee S. B. Kim N. Jo S. Hur Y. J. Lee J. Y. Cho J. H.. (2021). Mapping of a major QTL, qBK1Z, for bakanae disease resistance in rice. Plants 10 (3), 434. doi: 10.3390/plants10030434 33668736 PMC7996363

[B87] Lee J. H. Muhsin M. Atienza G. A. Kwak D. Y. Kim S. M. De Leon T. B.. (2010). Single nucleotide polymorphisms in a gene for translation initiation factor (*eIF4G*) of rice (*Oryza sativa*) associated with resistance to Rice tungro spherical virus. Mol. Plant Microbe Interact. 23 (1), 29–38. doi: 10.1094/MPMI-23-1-0029 19958136

[B88] Lee S. K. Song M. Y. Seo Y. S. Kim H. K. Ko S. Cao P. J.. (2009). Rice *Pi5*-mediated resistance to *Magnaporthe oryzae* requires the presence of two coiled-coil–nucleotide-binding–leucine-rich repeat genes. Genetics 181 (4), 1627–1638. doi: 10.1534/genetics.108.099226 19153255 PMC2666525

[B89] Leelagud P. Kongsila S. Vejchasarn P. Darwell K. Phansenee Y. Suthanthangjai A.. (2020). Genetic diversity of Asian rice gall midge based on *mtCOI* gene sequences and identification of a novel resistance locus *gm12* in rice cultivar MN62M. Mol. Biol. Rep. 47, 4273–4283. doi: 10.1007/s11033-020-05546-9 32468258

[B90] Li W. Chern M. Yin J. Wang J. Chen X. (2019). Recent advances in broad-spectrum resistance to the rice blast disease. Curr. Opin. Plant Biol. 50, 114–120. doi: 10.1016/j.pbi.2019.03.015 31163394

[B91] Li W. Zhu Z. Chern M. Yin J. Yang C. Ran L.. (2017). A natural allele of a transcription factor in rice confers broad-spectrum blast resistance. Cell 170 (1), 114–126. doi: 10.1016/j.cell.2017.06.008 28666113

[B92] Lin F. Chen S. Que Z. Wang L. Liu X. Pan Q. (2007). The blast resistance gene *Pi37* encodes a nucleotide binding site–leucine-rich repeat protein and is a member of a resistance gene cluster on rice chromosome 1. Genetics 177 (3), 1871–1880. doi: 10.1534/genetics.107.080648 17947408 PMC2147969

[B93] Lin S.-C. Li Y. Hu F.-Y. Wang C.-L. Kuang Y.-H. Sung C.-L.. (2022). Effect of nitrogen fertilizer on the resistance of rice near-isogenic lines with BPH resistance genes. Bot. Stud. 63, 16. doi: 10.1186/s40529-022-00347-8 35604579 PMC9127031

[B94] Liu X. Lin F. Wang L. Pan Q. (2007). The in *silico* map-based cloning of *Pi36*, a rice coiled-coil–nucleotide-binding site–leucine-rich repeat gene that confers race-specific resistance to the blast fungus. Genetics 176 (4), 2541–2549. doi: 10.1534/genetics.107.075465 17507669 PMC1950653

[B95] Liu W. Liu J. Ning Y. Ding B. Wang X. Wang Z.. (2013). Recent progress in understanding PAMP-and effector-triggered immunity against the rice blast fungus *Magnaporthe oryzae* . Mol. Plant 6 (3), 605–620. doi: 10.1093/mp/sst015 23340743

[B96] Liu Y. Liu B. Zhu X. Yang J. Bordeos A. Wang G.. (2013). Fine-mapping and molecular marker development for Pi56 (t), a NBS-LRR gene conferring broad-spectrum resistance to *Magnaporthe oryzae* in rice. Theoret. Appl. Genet. 126, 985–998. doi: 10.1007/s00122-012-2031-3 23400829

[B97] Liu Y. Wu H. Chen H. Liu Y. He J. Kang H.. (2015). A gene cluster encoding lectin receptor kinases confers broad-spectrum and durable insect resistance in rice. Nat. Biotechnol. 33 (3), 301–305. doi: 10.1038/nbt.3069 25485617

[B98] Liu Q. Yuan M. Zhou Y. A. N. Li X. Xiao J. Wang S. (2011). A paralog of the MtN3/saliva family recessively confers race-specific resistance to *Xanthomonas oryzae* in rice. Plant Cell Environ. 34 (11), 1958–1969. doi: 10.1111/j.1365-3040.2011.02391.x 21726237

[B99] Liu Z. Zhu Y. Shi H. Qiu J. Ding X. Kou Y. (2021). Recent progress in rice broad-spectrum disease resistance. Int. J. Mol. Sci. 22 (21), 11658. doi: 10.3390/ijms222111658 34769087 PMC8584176

[B100] Ma J. Lei C. Xu X. Hao K. Wang J. Cheng Z.. (2015). *Pi64*, encoding a novel CC-NBS-LRR protein confers resistance to leaf and neck blast in rice. Mol. Plant Microbe Interact. 28 (5), 558–568. doi: 10.1094/MPMI-11-14-0367-R 25650828

[B101] Ma L. Yu Y. Li C. Wang P. Liu K. Ma W.. (2022). Genome-wide association study identifies a rice panicle blast resistance gene *Pb3* encoding NLR protein. Int. J. Mol. Sci. 23, 14032. doi: 10.3390/ijms232214032 36430507 PMC9698523

[B102] Macovei A. Sevilla N. R. Cantos C. Jonson G. B. Slamet-Loedin I. Čermák T.. (2018). Novel alleles of rice *eIF4G* generated by CRISPR/Cas9-targeted mutagenesis confer resistance to Rice tungro spherical virus. Plant Biotechnol. J. 16 (11), 1918–1927. doi: 10.1111/pbi.12927 29604159 PMC6181218

[B103] Matsumoto K. Ota Y. Seta S. Nakayama Y. Ohno T. Mizobuchi R.. (2017). Identification of QTLs for rice brown spot resistance in backcross inbred lines derived from a cross between Koshihikari and CH45. Breed. Sci. 67 (5), 540–543. doi: 10.1270/jsbbs.17057 29398949 PMC5790048

[B104] McCouch S. R. Teytelman L. Xu Y. Lobos K. B. Clare K. Walton M.. (2002). Development and mapping of 2240 new SSR markers for rice (*Oryza sativa* L.). DNA Res. 9 (6), 199–207. doi: 10.1093/dnares/9.6.199 12597276

[B105] McKenzie K. S. Johnson C. W. Tseng S. T. Oster J. J. Brandon D. M. (1994). Breeding improved rice cultivars for temperate regions: a case study. Aust. J. Exp. Agric. 34 (7), 897–905. doi: 10.1071/EA9940897

[B106] Meng X. Xiao G. Telebanco-Yanoria M. J. Siazon P. M. Padilla J. Opulencia R.. (2020). The broad-spectrum rice blast resistance (R) gene *Pita2* encodes a novel R protein unique from *Pita* . Rice 13 (19). doi: 10.1186/s12284-020-00377-5 PMC7070119 32170462

[B107] Mizobuchi R. Fukuoka S. Tsushima S. Yano M. Sato H. (2016). QTLs for resistance to major rice diseases exacerbated by global warming: brown spot, bacterial seedling rot, and bacterial grain rot. Rice 9 (1), 1–12. doi: 10.1186/s12284-016-0095-4 27178300 PMC4870548

[B108] Molla K. A. Karmakar S. Molla J. Bajaj P. Varshney R. K. Datta. (2020). Understanding sheath blight resistance in rice: the road behind and the road ahead. Plant Biotechnol. J. 18 (4), 895–915. doi: 10.1111/pbi.13312 31811745 PMC7061877

[B109] Musonerimana S. Bez C. Licastro D. Habarugira G. Bigirimana J. Venturi V. (2020). Pathobiomes revealed that *Pseudomonas fuscovaginae* and *Sarocladium oryzae* are independently associated with rice. Microb. Ecol. 80, 627–642. doi: 10.1007/s00248-020-01529-2 32474660

[B110] Nadeem M. A. Nawaz M. A. Shahid M. Q. Doğan Y. Comertpay G. Yıldız M. (2018). DNA molecular markers in plant breeding: current status and recent advancements in genomic selection and genome editing. Biotechnol. Biotechnol. Equip. 32 (2), 261–285. doi: 10.1080/13102818.2017.1400401

[B111] Naik S. B. Divya D. Sahu N. Sundaram R. M. Sarao P. S. Singh K.. (2018). A new gene *Bph33* (t) conferring resistance to brown planthopper (BPH), *Nilaparvata lugens* (Stål) in rice line RP2068-18-3-5. Euphytica 214, 1–12. doi: 10.1007/s10681-018-2131-5

[B112] Ndjiondjop M. N. Albar L. Fargette D. Fauquet C. Ghesquière A. (1999). The genetic basis of high resistance to rice yellow mottle virus (RYMV) in cultivars of two cultivated rice species. Plant Dis. 83 (10), 931–935. doi: 10.1094/PDIS.1999.83.10.931 30841075

[B113] Neelam K. Kumar K. Kaur A. Kishore A. Kaur P. Babbar A.. (2022). High-resolution mapping of the quantitative trait locus (QTLs) conferring resistance to false smut disease in rice. J. Appl. Genet. 63 (1), 35–45. doi: 10.1007/s13353-021-00659-8 34535887

[B114] Ogawa T. Yamamoto T. Khush G. S. Mew T. W. (1991). Breeding of near-isogenic lines of rice with single genes for resistance to bacterial blight pathogen (*Xanthomonas campestris* pv. *oryzae*). Jpn. J. Breed. 41 (3), 523–529. doi: 10.1270/jsbbs1951.41.523

[B115] Okuyama Y. Kanzaki H. Abe A. Yoshida K. Tamiru M. Saitoh H.. (2011). A multifaceted genomics approach allows the isolation of the rice *Pia*-blast resistance gene consisting of two adjacent NBS-LRR protein genes. Plant J. 66 (3), 467–479. doi: 10.1111/j.1365-313X.2011.04502.x 21251109

[B116] Oliva R. Ji C. Atienza-Grande G. Huguet-Tapia J. C. Perez-Quintero A. Li T.. (2019). Broad-spectrum resistance to bacterial blight in rice using genome editing. Nat. Biotechnol. 37 (11), 1344–1350. doi: 10.1038/s41587-019-0267-z 31659337 PMC6831514

[B117] Orjuela J. Deless E. T. Kolade O. Chéron S. Ghesquière A. Albar L. (2013). A recessive resistance to rice yellow mottle virus is associated with a rice homolog of the *CPR5* gene, a regulator of active defense mechanisms. Mol. Plant Microbe Interact. 26 (12), 1455–1463. doi: 10.1094/MPMI-05-13-0127-R 23944999

[B118] Ortega L. Rojas C. M. (2021). Bacterial Panicle Blight and *Burkholderia glumae*: From pathogen biology to disease control. Phytopathology 111 (5), 772–778. doi: 10.1094/PHYTO-09-20-0401-RVW 33206007

[B119] Oster J. J. (1992). Reaction of a resistant breeding line and susceptible California rice cultivars to *Sclerotium oryzae* . Plant Dis. 76 (7), 740–744. doi: 10.1094/PD-76-0740

[B120] Pathak M. D. Khan Z. R. (1994). Insect Pests of Rice (Philippines: International Rice Research Institute).

[B121] Pennisi E. (2010). Armed and dangerous. Science 327, 804–805. doi: 10.1126/science.327.5967.804 20150482

[B122] Phi C. N. Fujita D. Yamagata Y. Yoshimura A. Yasui H. (2019). High-resolution mapping of *GRH6*, a gene from *Oryza nivara* (Sharma et Shastry) conferring resistance to green rice leafhopper (*Nephotettix cincticeps* Uhler). Breed. Sci. 69 (3), 439–446. doi: 10.1270/jsbbs.19029 31598076 PMC6776147

[B123] Pidon H. Chéron S. Ghesquière A. Albar L. (2020). Allele mining unlocks the identification of RYMV resistance genes and alleles in African cultivated rice. BMC Plant Biol. 20 (1), 1–14. doi: 10.1186/s12870-020-02433-0 32429875 PMC7236528

[B124] Pidon H. Ghesquière A. Chéron S. Issaka S. Hébrard E. Sabot F.. (2017). Fine mapping of *RYMV3*: a new resistance gene to Rice yellow mottle virus from Oryza glaberrima. Theor. Appl. Genet. 130, 807–818. doi: 10.1007/s00122-017-2853-0 28144699

[B125] Pradhan S. K. Nayak D. K. Mohanty S. Behera L. Barik S. R. Pandit E.. (2015). Pyramiding of three bacterial blight resistance genes for broad-spectrum resistance in deepwater rice variety, Jalmagna. Rice 8, 19. doi: 10.1186/s12284-015-0051-8 26054243 PMC4489969

[B126] Pradhan S. K. Barik S. R. Nayak D. K. Pradhan A. Pandit E. Nayak. (2020). Genetics, molecular mechanisms and deployment of bacterial blight resistance genes in rice. Crit. Rev. Plant Sci. 39 (4), 360–385. doi: 10.1080/07352689.2020.1801559

[B127] Qin J. Wang C. Wang L. Zhao S. Wu J. (2019). Defense and counter-defense in rice–virus interactions. Phytopathol. Res. 1, 1–6. doi: 10.1186/s42483-019-0041-7

[B128] Qiu Y. Guo J. Jing S. Zhu L. He G. (2012). Development and characterization of *japonica* rice lines carrying the brown planthopper-resistance genes *BPH12* and *BPH6* . Theor. Appl. Genet. 124, 485–494. doi: 10.1007/s00122-011-1722-5 22038433

[B129] Qu S. Liu G. Zhou B. Bellizzi M. Zeng L. Dai L.. (2006). The broad-spectrum blast resistance gene *Pi9* encodes a nucleotide-binding site–leucine-rich repeat protein and is a member of a multigene family in rice. Genetics 172 (3), 1901–1914. doi: 10.1534/genetics.105.044891 16387888 PMC1456263

[B130] Rao Y. Dong G. Zeng D. Hu J. Zeng L. Gao Z.. (2010). Genetic analysis of leaffolder resistance in rice. J. Genet. Genomics 37 (5), 325–331. doi: 10.1016/S1673-8527(09)60050-3 20513633

[B131] Ren J. Gao F. Wu X. Lu X. Zeng L. Lv J.. (2016). *Bph32*, a novel gene encoding an unknown SCR domain-containing protein, confers resistance against the brown planthopper in rice. Sci. Rep. 6 (1), 37645. doi: 10.1038/srep37645 27876888 PMC5120289

[B132] Rosas J. E. Martínez S. Blanco P. Perez de Vida F. Bonnecarrère V. Mosquera G.. (2018). Resistance to multiple temperate and tropical stem and sheath diseases of rice. Plant Genome 11 (1), 170029. doi: 10.3835/plantgenome2017.03.0029 PMC12810057 29505639

[B133] Sama V. S. A. K. Rawat N. Sundaram R. M. Himabindu K. Naik B. S. Viraktamath B. C.. (2014). A putative candidate for the recessive gall midge resistance gene *gm3* in rice identified and validated. Theor. Appl. Genet. 127, 113–124. doi: 10.1007/s00122-013-2205-7 24145853

[B134] Savary S. Willocquet L. Pethybridge S. J. Esker P. McRoberts N. Nelson A. (2019). The global burden of pathogens and pests on major food crops. Nat. Ecol. Evol. 3 (3), 430–439. doi: 10.1038/s41559-018-0793-y 30718852

[B135] Schneider P. Asch F. (2020). Rice production and food security in Asian Mega deltas—A review on characteristics, vulnerabilities and agricultural adaptation options to cope with climate change. J. Agron. Crop Sci. 206 (4), 491–503. doi: 10.1111/jac.12415

[B136] Seck P. A. Diagne A. Mohanty S. Wopereis M. C. (2012). Crops that feed the world 7: Rice. Food Sec. 4, 7–24. doi: 10.1007/s12571-012-0168-1

[B137] Shang J. Tao Y. Chen X. Zou Y. Lei C. Wang J.. (2009). Identification of a new rice blast resistance gene, *Pid3*, by genome-wide comparison of paired nucleotide-binding site–leucine-rich repeat genes and their pseudogene alleles between the two sequenced rice genomes. Genetics 182 (4), 1303–1311. doi: 10.1534/genetics.109.102871 19506306 PMC2728867

[B138] Shi S. Wang H. Nie L. Tan D. I. Zhou C. Zhang Q.. (2021). *Bph30* confers resistance to brown planthopper by fortifying sclerenchyma in rice leaf sheaths. Mol. Plant 14, 1714–1732. doi: 10.1016/j.molp.2021.07.004 34246801

[B139] Si W. Yuan Y. Huang J. Zhang X. Zhang Y. Zhang Y.. (2015). Widely distributed hot and cold spots in meiotic recombination as shown by the sequencing of rice F2 plants. New Phytol. 206 (4), 1491–1502. doi: 10.1111/nph.13319 25664766

[B140] Singh P. Verma R. L. Singh R. S. Singh R. P. Singh H. B. Arsode P.. (2020). “Biotic stress management in rice (*Oryza sativa* L.) through conventional and molecular approaches,” in New Frontiers in Stress Management For Durable Agriculture. Eds. Rakshit A. Singh H. Singh A. Singh U. Fraceto L. (Singapore: Springer), 609–644.

[B141] Song W. Y. Wang G. L. Chen L. L. Kim H. S. Pi L. Y. Holsten T.. (1995). A receptor kinase-like protein encoded by the rice disease resistance gene, *Xa21* . Science 270 (5243), 1804–1806. doi: 10.1126/science.270.5243.1804 8525370

[B142] Su J. Wang W. Han J. Chen S. Wang C. Zeng L.. (2015). Functional divergence of duplicated genes results in a novel blast resistance gene *Pi50* at the *Pi2/9* locus. Theoret. Appl. Genet. 128, 2213–2225. doi: 10.1007/s00122-015-2579-9 26183036

[B143] Sun X. Cao Y. Yang Z. Xu C. Li X. Wang S.. (2004). *Xa26*, a gene conferring resistance to *Xanthomonas oryzae* pv. *oryzae* in rice, encodes an LRR receptor kinase-like protein. Plant J. 37 (4), 517–527. doi: 10.1046/j.1365-313X.2003.01976.x 14756760

[B144] Sun L. Su C. Wang C. Zhai H. Wan J. (2005). Mapping of a major resistance gene to the brown planthopper in the rice cultivar Rathu Heenati. Breed. Sci. 55 (4), 391–396. doi: 10.1270/jsbbs.55.391

[B145] Takahashi A. Hayashi N. Miyao A. Hirochika H. (2010). Unique features of the rice blast resistance *Pish* locus revealed by large scale retrotransposon-tagging. BMC Plant Biol. 10, 1–14. doi: 10.1186/1471-2229-10-175 20707904 PMC3017791

[B146] Tamura Y. Hattori M. Yoshioka H. Yoshioka M. Takahashi A. Wu J. (2014). Map-based cloning and characterization of a brown planthopper resistance gene *BPH26* from *Oryza sativa* L. ssp. *indica* cultivar ADR52. Sci. Rep. 4 (1), 1–8. doi: 10.1038/srep05872 PMC5376202 25076167

[B147] Tang D. Wu W. Li W. Lu H. Worland A. J. (2000). Mapping of QTLs conferring resistance to bacterial leaf streak in rice. Theor. Appl. Genet. 101, 286–291. doi: 10.1007/s001220051481

[B148] Telebanco-Yanoria M. J. Koide Y. Fukuta Y. Imbe T. Kato H. Tsunematsu H.. (2010). Development of near-isogenic lines of *Japonica*-type rice variety Lijiangxintuanheigu as differentials for blast resistance. Breed. Sci. 60 (5), 629–638. doi: 10.1270/jsbbs.60.629

[B149] Telebanco-Yanoria M. J. Koide Y. Fukuta Y. Imbe T. Tsunematsu H. Kato H.. (2011). A set of near-isogenic lines of *Indica*-type rice variety CO 39 as differential varieties for blast resistance. Mol. Breed. 27, 357–373. doi: 10.1007/s11032-010-9437-x

[B150] Thiémélé D. Boisnard A. Ndjiondjop M. N. Chéron S. Séré Y. Aké S.. (2010). Identification of a second major resistance gene to Rice yellow mottle virus, *RYMV2*, in the African cultivated rice species, *O. glaberrima* . Theor. Appl. Genet. 121, 169–179. doi: 10.1007/s00122-010-1300-2 20198467

[B151] Tian D. Wang J. Zeng X. Gu K. Qiu C. Yang X. (2014). The rice TAL effector–dependent resistance protein *XA10* triggers cell death and calcium depletion in the endoplasmic reticulum. Plant Cell 26 (1), 497–515. doi: 10.1105/tpc.113.119255 24488961 PMC3963592

[B152] Velásquez A. C. Castroverde C. D. M. He S. Y. (2018). Plant–pathogen warfare under changing climate conditions. Curr. Biol. 28 (10), R619–R634. doi: 10.1016/j.cub.2018.03.054 29787730 PMC5967643

[B153] Wang Y. Cao L. Zhang Y. Cao C. Liu F. Huang F.. (2015). Map-based cloning and characterization of *BPH29*, a B3 domain-containing recessive gene conferring brown planthopper resistance in rice. J. Exp. Bot. 66 (19), 6035–6045. doi: 10.1093/jxb/erv318 26136269 PMC4566989

[B154] Wang C. Chen S. Feng A. Su J. Wang W. Feng J.. (2021). *Xa7*, a small orphan gene harboring promoter trap for *AvrXa7*, leads to the durable resistance to *Xanthomonas oryzae* pv. *oryzae* . Rice 14 (1), 1–16. doi: 10.1186/s12284-021-00490-z 34056673 PMC8165051

[B155] Wang H. M. Chen J. Shi Y. F. Pan G. Shen H. C. Wu J. L. (2012). Development and validation of CAPS markers for marker-assisted selection of rice blast resistance gene *Pi25* . Acta Agron. Sin. 38 (11), 1960–1968. doi: 10.3724/SP.J.1006.2012.01960

[B156] Wang Q. Liu Y. He J. Zheng X. Hu J. Liu Y.. (2014). *STV11* encodes a sulphotransferase and confers durable resistance to rice stripe virus. Nat. Commun. 5, 4768. doi: 10.1038/ncomms5768 25203424 PMC4164775

[B157] Wang Z. X. Yano M. Yamanouchi U. Iwamoto M. Monna L. Hayasaka H.. (1999). The *Pib* gene for rice blast resistance belongs to the nucleotide binding and leucine-rich repeat class of plant disease resistance genes. Plant J. 19 (1), 55–64. doi: 10.1046/j.1365-313X.1999.00498.x 10417726

[B158] Wang C. Zhang X. Fan Y. Gao Y. Zhu Q. Zheng C.. (2015). *XA23* is an executor R protein and confers broad-spectrum disease resistance in rice. Mol. Plant 8 (2), 290–302. doi: 10.1016/j.molp.2014.10.010 25616388

[B159] Webb K. M. Ona I. Bai J. Garrett K. A. Mew T. Cruz C. M. V.. (2010). A benefit of high temperature: increased effectiveness of a rice bacterial blight disease resistance gene. New Phytol. 185, 568–576. doi: 10.1111/j.1469-8137.2009.03076.x 19878463

[B160] Wu Y. Xiao N. Chen Y. Yu L. Pan C. Li Y.. (2019). Comprehensive evaluation of resistance effects of pyramiding lines with different broad-spectrum resistance genes against *Magnaporthe oryzae* in rice (*Oryza sativa* L.). Rice 12, 1–13. doi: 10.1186/s12284-019-0264-3 30825053 PMC6397272

[B161] Xie X. Chen Z. Cao J. Guan H. Lin D. Li C.. (2014). Toward the positional cloning of *qBlsr5a*, a QTL underlying resistance to bacterial leaf streak, using overlapping sub-CSSLs in rice. PloS One 9 (4), e95751. doi: 10.1371/Journal.Pone.0095751 24752581 PMC3994123

[B162] Xie Z. Yan B. Shou J. Tang J. Wang X. Zhai K.. (2019). A nucleotide-binding site-leucine-rich repeat receptor pair confers broad-spectrum disease resistance through physical association in rice. Philos. Trans. R. Soc B. 374 (1767), 20180308. doi: 10.1098/rstb.2018.0308 PMC6367147 30967012

[B163] Xie X. Zheng Y. Lu L. Yuan J. Hu J. Bu S.. (2021). Genome-wide association study of QTLs conferring resistance to bacterial leaf streak in rice. Plants 10 (10), 2039. doi: 10.3390/plants10102039 34685848 PMC8541590

[B164] Xing J. Zhang D. Yin F. Zhong Q. Wang B. Xiao S.. (2021). Identification and fine-mapping of a new bacterial blight resistance gene, Xa47 (t), in G252, an introgression line of Yuanjiang common wild rice (*Oryza rufipogon*). Plant Dis. 105 (12), 4106–4112. doi: 10.1094/PDIS-05-21-0939-RE 34261357

[B165] Xu X. Hayashi N. Wang C. T. Fukuoka S. Kawasaki S. Takatsuji H.. (2014). Rice blast resistance gene Pikahei-1 (t), a member of a resistance gene cluster on chromosome 4, encodes a nucleotide-binding site and leucine-rich repeat protein. Mol. Breed. 34, 691–700. doi: 10.1007/s11032-014-0067-6

[B166] Yan L. Luo T. Huang D. Wei M. Ma Z. Liu C.. (2023). Recent advances in molecular mechanism and breeding utilization of brown planthopper resistance genes in rice: An integrated review. Int. J. Mol. Sci. 24 (15), 12061. doi: 10.3390/ijms241512061 37569437 PMC10419156

[B167] Yang M. Lin J. Cheng L. Zhou H. Chen S. Liu F.. (2020). Identification of a novel planthopper resistance gene from wild rice (*Oryza rufipogon* Griff.). Crop J. 8 (6), 1057–1070. doi: 10.1016/j.cj.2020.03.011

[B168] Yoshimura S. Yamanouchi U. Katayose Y. Toki S. Wang Z. X. Kono I.. (1998). Expression of *Xa1*, a bacterial blight-resistance gene in rice, is induced by bacterial inoculation. Proc. Natl. Acad. Sci. 95 (4), 1663–1668. doi: 10.1073/pnas.95.4.1663 9465073 PMC19140

[B169] Yu Y. Ma L. Wang X. Zhao Z. Wang W. Fan Y.. (2022). Genome-wide association study identifies a rice panicle blast resistance gene, *Pb2*, encoding. Int. J. Mol. Sci. 23 (10), 5668. doi: 10.3390/ijms23105668 35628477 PMC9145240

[B170] Yuan B. Zhai C. Wang W. Zeng X. Xu X. Hu H.. (2011). The *Pik-p* resistance to *Magnaporthe oryzae* in rice is mediated by a pair of closely linked CC-NBS-LRR genes. Theoret. Appl. Genet. 122, 1017–1028. doi: 10.1007/s00122-010-1506-3 21153625

[B171] Zampieri E. Volante A. Marè C. Orasen G. Desiderio F. Biselli C.. (2023). Marker-assisted pyramiding of blast-resistance genes in a *japonica* elite rice cultivar through forward and background selection. Plants 12, 757. doi: 10.3390/plants12040757 36840105 PMC9963729

[B172] Zarbafi S. S. Ham J. H. (2019). An overview of rice QTLs associated with disease resistance to three major rice diseases: blast, sheath blight, and bacterial panicle blight. Agronomy 9 (4), 177. doi: 10.3390/agronomy9040177

[B173] Zhai C. Lin F. Dong Z. He X. Yuan B. Zeng X.. (2011). The isolation and characterization of *Pik*, a rice blast resistance gene which emerged after rice domestication. New Phytol. 189 (1), 321–334. doi: 10.1111/j.1469-8137.2010.03462.x 21118257

[B174] Zhang L. Nakagomi Y. Endo T. Teranishi M. Hidema J. Sato S.. (2018). Divergent evolution of rice blast resistance *Pi54* locus in the genus *Oryza* . Rice 11, 1–13. doi: 10.1186/s12284-018-0256-8 30519841 PMC6281543

[B175] Zhao Y. Huang J. Wang Z. Jing S. Wang Y. Ouyang Y.. (2016). Allelic diversity in an NLR gene *BPH9* enables rice to combat planthopper variation. Proc. Natl. Acad. Sci. 113 (45), 12850–12855. doi: 10.1073/pnas.1614862113 27791169 PMC5111712

[B176] Zhao H. Wang X. Jia Y. Minkenberg B. Wheatley M. Fan J.. (2018). The rice blast resistance gene *Ptr* encodes an atypical protein required for broad-spectrum disease resistance. Nat. Commun. 9 (1) 2039. doi: 10.1038/s41467-018-04369-4 29795191 PMC5966436

[B177] Zhou X. G. (2019). “Sustainable strategies for managing bacterial panicle blight in rice,” in Protecting Rice Grains in The Post-Genomic Era. Ed. Jia Y. (London, UK: IntechOpen), 67–80.

[B178] Zhou B. Qu S. Liu G. Dolan M. Sakai H. Lu G.. (2006). The eight amino-acid differences within three leucine-rich repeats between *Pi2* and *Piz-t* resistance proteins determine the resistance specificity to *Magnaporthe grisea* . Mol. Plant Microbe Interact. 19 (11), 1216–1228. doi: 10.1094/MPMI-19-1216 17073304

